# Mesenchymal stem/stromal cell senescence in aging and regenerative medicine: a systematic review

**DOI:** 10.1007/s10522-026-10501-5

**Published:** 2026-09-25

**Authors:** Letícia Odaguiri Watanabe, Luana Roleto Cardoso, Johnny Carvalho da Silva, Larissa Di Carvalho, Vitoria Castro, Juliana Lott Carvalho, Eliete Guerra, Taia Maria Berto Rezende

**Affiliations:** 1 Postgraduate Program in Health Sciences, Faculty of Health Sciences, University of Brasília, Brasília, Brazil; 2 Postgraduate Program in Dentistry, Faculty of Health Sciences, University of Brasília, Brasília, Brazil; 3 Laboratory of Interdisciplinary Biosciences, Faculty of Medicine, University of Brasília, Brasília, Brazil; 4 Postgraduate Program in Genomic Sciences and Biotechnology, Catholic University of Brasília, Brasília, Brazil; 5 Department of Dentistry, Faculty of Health Sciences, University of Brasília, Brasília, Brazil

**Keywords:** Stem cells, Cellular senescence, Aging, Tissue regeneration, Regenerative medicine, Tissue engineering

## Introduction

Mesenchymal stem/stromal cells (MSCs) have emerged as a promising cell source for tissue engineering and cell-based therapies due to their capacity for self-renewal, multilineage differentiation, and immunomodulatory effects (Pittenger et al. 1999). These cells can be isolated from a wide range of human tissues, including bone marrow (BM-MSCs), adipose tissue (AD-MSCs), dental tissues, such as dental pulp stem cells (DPSCs) and periodontal ligament stem cells (PDLSCs), as well as umbilical cord cells (UC-MSCs), including Wharton’s jelly–derived MSCs (UC-MSCs-WJ) and umbilical cord blood–derived MSCs (UC-MSCs-CB) (Kern et al. 2006; Heo et al. 2016). The therapeutic potential of MSCs is closely linked to their functional properties, including their capacity to migrate to sites of injury, modulate immune responses, and promote tissue repair predominantly through paracrine signaling, in addition to limited proliferative and differentiation potential (Zuk et al. 2001).

Despite their considerable therapeutic potential, MSCs are susceptible to cellular senescence, a complex process characterized by irreversible cell cycle arrest, accompanied by morphological and metabolic changes, such as the acquisition of a senescence-associated secretory phenotype (SASP) (Kirkland and Tchkonia 2017). Key functional impairments include reduced proliferative capacity, decreased migratory ability, and compromised osteogenic, adipogenic, and chondrogenic differentiation — standard indicators of MSC multipotency (Wenjing et al. 2024). In addition, senescence is associated with the development of SASP, characterized by the sustained release of pro-inflammatory mediators, which impair tissue regeneration and perpetuate a chronic inflammatory microenvironment, further exacerbating MSC functional decline (Coppé et al. 2008; Su et al. 2020). In experimental MSC research, senescence has been investigated in the context of chronological aging, as well as through in vitro models of biological aging, including replicative senescence induced by repeated cell passaging and stress-induced senescence triggered by inflammatory stimuli (Gruber et al. 2012; Feng et al. 2014a; Choudhery et al. 2014). However, the extent to which these distinct contexts of senescence differentially affect MSC functional outcomes remains insufficiently synthesized. Understanding these changes is important for the clinical translation of MSC-based therapies, as the administration of senescent cells may not only reduce therapeutic efficacy but also pose safety concerns due to their pro-inflammatory secretome.

Cellular senescence is increasingly recognized as a relevant feature of ageing, but important knowledge gaps remain regarding its biological significance, including how senescent cells influence tissue function and regeneration and how senescence-related changes differ across cellular and environmental contexts (Rattan 2024). Consistent with these gaps, recent work in biogerontology has identified the role of senescent cells as an important open question in ageing science, particularly regarding when senescent cells are beneficial or detrimental (Talay et al. 2026). In this context, understanding senescence in MSCs may provide important insights into age-associated changes in regenerative capacity and into the potential limitations of using these cells for regenerative therapies.

Although numerous studies have examined senescence-associated changes in MSCs, findings remain fragmented and difficult to compare across experimental models and outcome measures. This inconsistency stems from the wide variety of markers and methods that are used to confirm the senescent state, including senescence-associated β-galactosidase activity, the upregulation of cell cycle-related proteins (p16, p21, and p53), telomere length or activity (Horibe et al. 2014; Zhang et al. 2024). Beyond differences in senescence models and markers, the tissue source of MSCs represents an additional layer of biological variability that may influence senescence-associated functional outcomes (Ma et al. 2019).

Given the heterogeneity of experimental models, senescence contexts, and MSC tissue sources, a comprehensive synthesis of the available evidence is warranted. Rather than focusing on a single tissue source or senescence model, this systematic review integrates findings from multiple MSC origins and aging contexts to provide an overarching understanding of how cellular senescence affects their functional properties. Therefore, this review aims to evaluate the effects of cellular senescence on key functional characteristics of mesenchymal stem cells derived from adipose tissue, bone marrow, dental pulp, periodontal ligament, Wharton’s jelly, and umbilical cord blood, considering chronological donor aging, serial in vitro passaging, and inflammatory stimulation as relevant senescence contexts.

## Methods

### Eligibility criteria

This review was conducted in accordance with PRISMA 2020 (Page et al. 2021) guidelines and the protocol were registered in PROSPERO (CRD42025638850). This systematic review addressed the question: “How does cellular senescence in stem cells from various tissues affect their regenerative potential and tissue repair mechanisms?” The eligibility criteria were defined based on the PECO framework (Population, Exposure, Comparison, and Outcome). The Population (P) included human MSCs derived from adipose tissue, bone marrow, dental pulp, periodontal ligament, umbilical cord blood, or Wharton’s jelly. The Exposure (E) was defined as cellular senescence induced by donor aging, serial passaging, or exposure to inflammatory stimuli. These contexts were compared against Comparators (C) consisting of non-senescent counterparts (derived from young donors, early-passage cultures, or unstimulated controls) used as comparators. These three senescence induction contexts were selected based on their distinct biological and translational relevance to MSC-based therapies: chronological aging reflects donor-related variability; serial passaging express in vitro expansion during manufacturing; and inflammatory stimuli mimic the chronic wound microenvironment where MSCs are therapeutically applied. Studies were required to assess at least one Outcome (O) related to regenerative capacity of the investigated MSCs, including proliferation, differentiation, migration, immune modulation, or in vivo repair outcomes and to confirm the cellular senescence state using at least one established marker (e.g., SA-β-gal activity, *CDKN2A* (p16)*/CDKN1A* (p21)*/TP53* (p53) expression, or telomere-related measures). Studies were only included if investigating primary, non-genetically modified stem or progenitor cells. No restrictions on language or publication year were applied. Reviews, conference materials, case reports, studies using immortalized or genetically modified cells, studies without a non-senescent comparator, or without confirmation of senescence were excluded.

### Information sources and search strategy

Searches were performed in MEDLINE/PubMed (via the National Library of Medicine), EMBASE (via Elsevier), Web of Science Core Collection (via Clarivate), and the Cochrane Database (via the Cochrane Library). Grey literature was searched using Google Scholar and ProQuest, and reference lists of included studies were manually screened. The initial search was conducted on 28 February 2025 and updated on 12 December 2025. Full search strategies for all databases are provided in Supplementary Table 1.

### Selection process

Duplicate records were removed using EndNote and Rayyan®. Titles and abstracts were independently screened by two reviewers, followed by full-text assessment for eligibility. Disagreements were resolved by a third reviewer. The study selection process is summarized using a PRISMA flow diagram (Page et al. 2021).

### Data collection process and data items

Data extraction was independently performed by two reviewers using a standardized form. The Deep-Seek AI-assisted tool was used to support extraction of general study characteristics and population data, with all outputs manually verified. Non-English articles were translated using automated translation software. Extracted data included MSC tissue source, senescence induction and confirmation methods, comparator group characteristics, and functional outcomes related to proliferation, differentiation, migration, immune response, and tissue repair. When multiple assays were reported for a given outcome, a predefined hierarchy was applied to select one representative measure for synthesis. Quantitative data not reported in the text were extracted from figures using PlotDigitizer (Version 3.1.6, 2025).

### Effect measures and synthesis methods

Due to substantial heterogeneity in senescence models, MSC sources, and outcome assessments, a quantitative meta-analysis was not performed. Results were synthesized narratively and summarized in tables organized by outcome category, senescence induction method, and MSC tissue source. Statistical significance was reported as described in the original studies; when comparative statistics were unavailable, outcomes were recorded as not reported (NR). Data handling and tabulation were performed using Jamovi Statistical Software (Version 2.6).

### Study risk of bias assessment

Risk of bias was independently assessed by two reviewers using a critical appraisal tool based on the PETRICCs guideline for in vitro studies and SYRCLE’s tool for in vivo studies (Hooijmans et al. 2014; Monteiro et al. 2025). Discrepancies were resolved by consensus with a third reviewer. Studies were classified as high, moderate, or low risk of bias based on predefined thresholds.

## Results

### Study selection

**Fig. 1:**
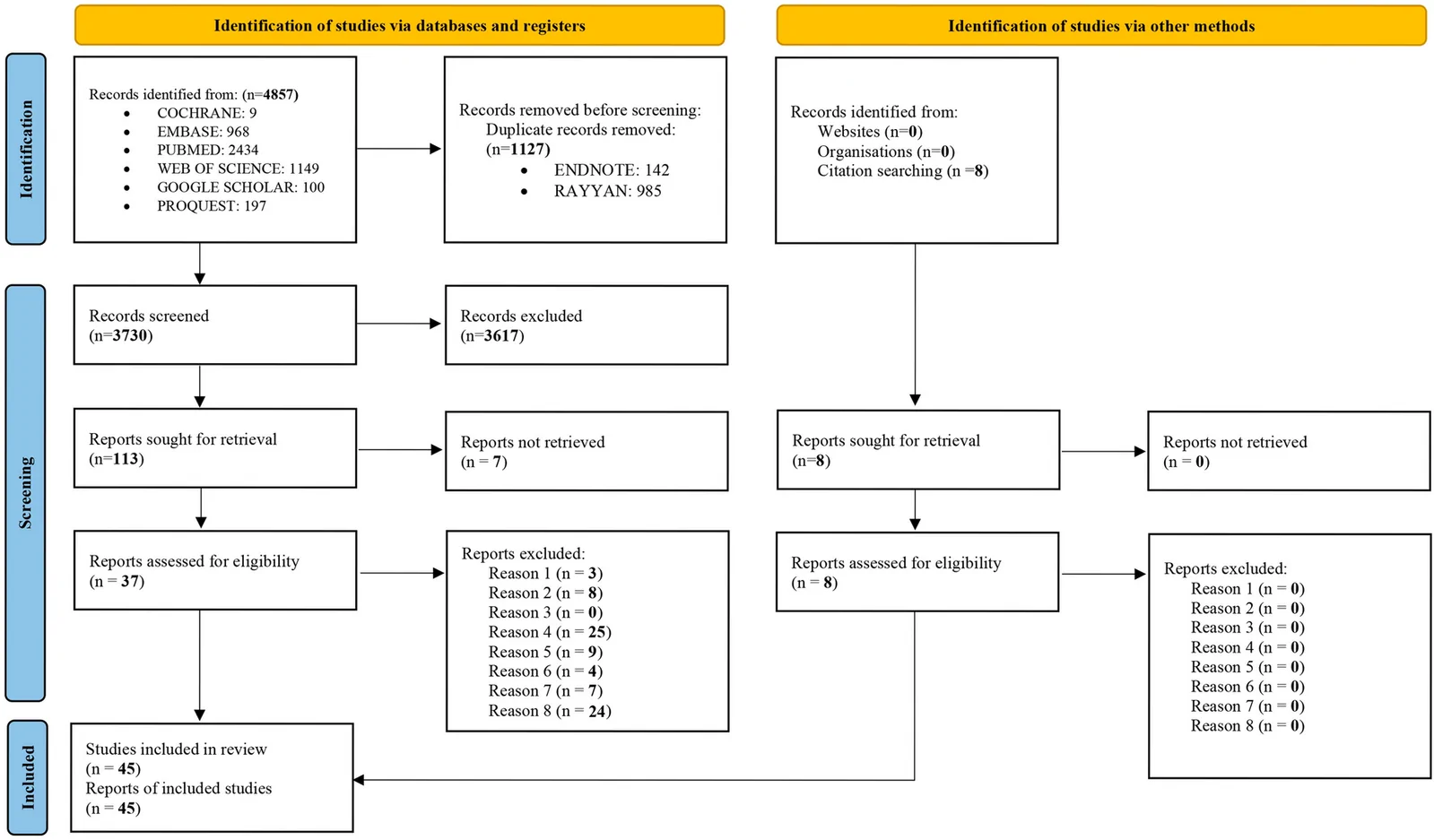
Preferred reporting items for systematic reviews and meta-analyses (PRISMA) flowchart

The study selection process is summarized in the PRISMA 2020 flow diagram (Fig. 1). Database searches identified 4,857 records, of which 1,127 duplicates were removed. After title and abstract screening, 113 full-text reports were assessed for eligibility. Of these, 76 were excluded based on predefined criteria (Fig. 1; Supplementary Table S2). An additional eight records were identified through citation searching and included. In total, 45 studies met the inclusion criteria and were included in the systematic review, with 35 in vitro studies and 10 combining in vitro and in vivo methodologies.

### Study characteristics

**Fig. 2:**
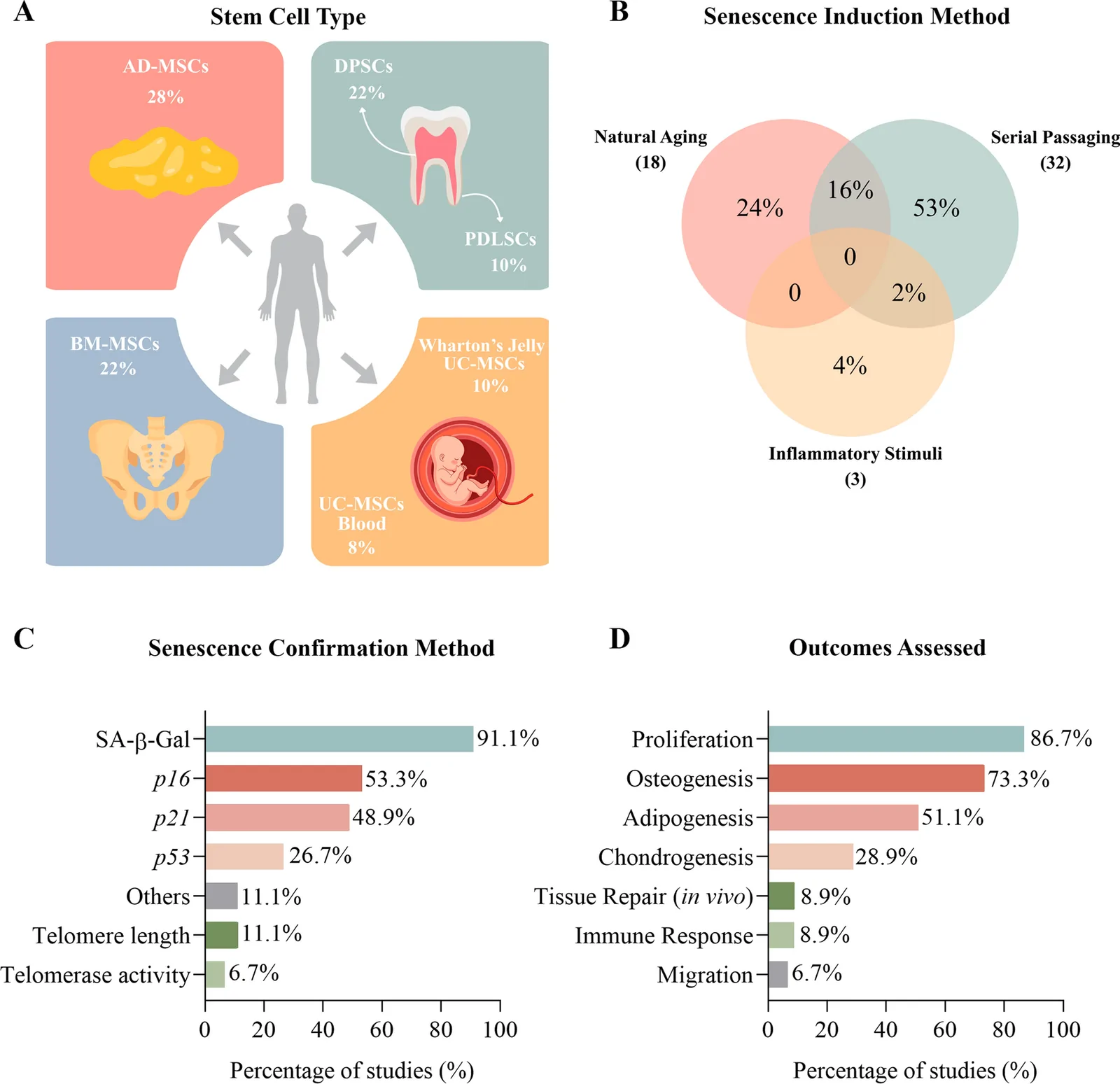
(**A**) Distribution of mesenchymal stem cell (MSC) types analyzed across studies. (**B**) Venn diagram of senescence induction methods. (**C**) Methods used to confirm cellular senescence. (**D**) Outcomes assessed in the included studies. In graphs C and D, the x-axis represents the number of included studies, with percentages indicate the proportion of studies evaluating each variable (n = 45)

A summary of study characteristics is presented in Fig. 2 and Table 1. Included studies were published between 2006 and 2025, with a higher concentration of publications observed from 2014 onwards. Most studies originated from China (n = 17; 37.8%), followed by South Korea (n = 5; 11.1%) and the United States (n = 4; 8.9%). AD-MSCs (28%), BM-MSCs (22%), and DPSCs (22%) were the most frequently studied cell types, while UC-MSCs and PDLSCs were less commonly evaluated (Fig. 2A). Serial passaging was the most common method used to induce senescence (n = 24, 53%), followed by chronological donor aging (n = 11, 24%); 16% (n = 7) of studies assessed both approaches. Inflammatory stimuli–induced senescence was investigated only in 3 studies (4%) (Fig. 2B). Senescence was most frequently confirmed using SA-β-gal staining (91.1%), followed by *CDKN2A* (p16) (53.3%)*/CDKN1A* (p21) (48.9%)*/TP53* (p53) (26.7%) expression (Fig. 2C). Proliferation (86.7%) and osteogenic (73.3%) differentiation were the most frequently assessed functional outcomes, whereas migration (6.7%), immune response (8.9%), and in vivo tissue repair (8.9%) were evaluated in relatively few studies (Fig. 2D).

**Table 1:** Characteristics of the included studies (n = 45)

Author /year/country	Study design	Stem cell source	Donor age (as reported)	n (donors)	Passage number	Senescence induction method	Senescence confirmation method	Outcomes assessed	Key findings (senescent group)
Alraies et al. (2017) UK	Experimental (in vitro)	DPSCs	18–30 years	3	NR (> 80 PD)	Serial Passaging	SA-β-Gal staining, *P16, P21* and *P53* expression	Proliferation and Differentiation (osteogenic and adipogenic)	*Short telomeres*: ↓proliferation ↓osteogenesis ↑adipogenesis ↓chondrogenesis
Alt et al. (2012) USA	Experimental (in vitro)	AD-MSCs	Group 1: <20 years Group 2: 30–40 years Group 3: >50 years	Group 1: 15 Group 2: 17 Group 3: 8	P1-P5	Chronological Aging	*P16* expression	Proliferation and Differentiation (osteogenic and adipogenic)	*Group 3*: ↓proliferation ↓osteogenesis ↓adipogenesis ↓chondrogenesis
Bertolo et al. (2016) Switzerland	Experimental (in vitro)	BM-MSCs	17–57 years (Mean: 34 ± 13 years)	7	P7-P11	Serial Passaging	SA-β-Gal staining	Proliferation and Differentiation (osteogenic, adipogenic and chondrogenic)	*Late-Passage*: ↓proliferation ↓osteogenesis ↑adipogenesis ↓chondrogenesis
Bonab et al. (2006) Iran	Experimental (in vitro)	BM-MSCs	2.5–63 years (Mean: 25 years)	11	P1-P10	Serial Passaging	Telomere shortening	Proliferation and Differentiation (osteogenic and adipogenic)	*Late-Passage*: ↓proliferation ↓osteogenesis ↓adipogenesis
Chen et al. (2024) China	Experimental (in vitro*, *in vivo)	DPSCs	16–70 years	25	P3-P5	Chronological Aging	SA-β-gal staining, *P21* and *P53* expression	Proliferation, Differentiation (osteogenic), Migration and Bone Repair (in vivo*)*	*Aged Group*: ↓proliferation ↓osteogenesis ↓migration; ↓bone repair
Cheng et al. (2011) China	Experimental (in vitro)	BM-MSCs UC-MSCs-WJ	Median: 19 years	5 4	P2-P13 P2-P25	Serial Passaging	SA-β-Gal staining	Proliferation and Differentiation (osteogenic and adipogenic)	*Late-Passage*: ↓proliferation ↓adipogenesis ↑osteogenesis
Choudhery et al. (2014) USA	Experimental (in vitro)	AD-MSCs	Group 1: <30 years (mean 25.5 ± 1.6) Group 2: 35–55 years (mean 46.4 ± 2.1) Group 3: >60 years (mean 66.0 ± 1.4)	Group 1: 8 Group 2: 10 Group 3: 11	P1-P3	Chronological Aging	SA-β-Gal staining, *P16* and *P21* expression	Proliferation and Differentiation (osteogenic, adipogenic and chondrogenic)	*Group 3*: ↓proliferation ↓osteogenesis →adipogenesis ↓chondrogenesis
Christodoulou et al. (2013) Greece	Experimental (in vitro)	UC-MSCs-WJ AD-MSCs	Fetal (full-term) 44 ± 11 years	5 3	P2-P20 P2-P10	Serial Passaging	SA-β-Gal staining	Proliferation and Differentiation (osteogenic)	*Late-Passage*: ↓proliferation ↓osteogenesis
Danisovic et al. (2017) Slovakia	Experimental (in vitro)	AD-MSCs	Mean: 34 years	5	P1-P30	Serial Passaging	Telomere activity	Proliferation	*Late-Passage*: ↓proliferation
Diomede et al. (2017) Italy	Experimental (in vitro)	DPSCs PDLSCs	NR	NR	P2-P15	Serial Passaging	SA-β-Gal staining, *P16* and *P21* expression	Proliferation and Differentiation (osteogenic and adipogenic)	*Late-Passage*: →proliferation →osteogenesis →adipogenesis
Feng et al. (2014a) China	Experimental (in vitro)	DPSCs	13–23 years	9	P3	Inflammatory Stimuli – LPS (1x, 3x, 6x)	SA-β-Gal staining	Proliferation	*LPS – 3x/6x*: ↓proliferation
Feng et al. (2014b) China	Experimental (in vitro)	DPSCs	5–12 years 12–20 years 20–35 years 35–50 years >50 years	33	P3	Chronological Aging	SA-β-gal staining and *p16* expression	Proliferation and Differentiation (osteogenic and adipogenic)	*Aged Group*: ↓proliferation ↓osteogenesis ↓adipogenesis
Feng et al. (2018)	Experimental (in vitro)	DPSC	13–23 years	9	P3	Inflammatory Stimuli – LPS (1x, 3x, 6x)	SA-β-Gal staining	Proliferation	*LPS – 3x/6x*: ↓proliferation
Figiel-Dabrowska et al. (2022) Poland	Experimental (in vitro)	AD-MSCs	NR	NR	P3-P22	Serial Passaging	SA-β-Gal staining	Proliferation	*Late-Passage*: ↓proliferation
Gao et al. (2023) China	Experimental (in vitro*, *in vivo)	BM-MSCs	NR	NR	P3-P4	Chronological Aging	SA-β-Gal staining, *P16* and *P21* expression	Proliferation and Cardiac Repair (in vivo)	*Aged Group*: ↓proliferation ↓cardiac repair (vs young group)
Govarthanan et al. (2025) India	Experimental (in vitro)	UC-MSCs-WJ	NR	NR	P3-P15	Serial Passaging	SA-β-Gal staining	Differentiation (osteogenic, adipogenic and chondrogenic)	*Late-Passage*: ↓osteogenesis ↓chondrogenesis
Gruber et al. (2012) USA	Experimental (in vitro)	AD-MSCs	30–73 years (Mean: 52.6 ± 12.8 years)	11	P1-P13	Chronological Aging and Serial Passaging	SA-β-Gal staining	Differentiation (osteogenic and chondrogenic)	*Aged Group and Late-Passage*: ↓proliferation →osteogenesis →chondrogenesis
Horibe et al. (2014) Japan	Experimental (in vitro*, *in vivo)	DPSCs	Young: 19–30 years Aged: 44–70 years	6	P5-P20	Chronological Aging and Serial Passaging	SA-β-gal staining, *p16*, *p21*, telomerase activity, telomere length	Proliferation, Differentiation (osteogenesis and adipogenesis), Pulp Regeneration and Immune Response	*Aged Group and Late-Passage*: ↓proliferation →osteogenesis →adipogenesis ↓pulp regeneration ↑IL-1β, IL-6, IL-8 and Groα
Hu et al. (2024) China	Experimental (in vitro)	PDLSCs	18–26 years	10	P4-P20	Serial Passaging	SA-β-Gal staining, *P16* and *P21* expression	Proliferation	*Late-Passage*: ↓proliferation
Huang et al. (2023) China	Experimental (in vitro)	BM-MSCs	NR	NR	P3-P12	Chronological Aging and Serial Passaging	SA-β-Gal staining, *P16* and *P21* expression	Proliferation and Immune Response	*Aged Group and Late-Passage*: ↓proliferation ↑TNF-α and IL-6 ↓IL-10
Iezzi et al. (2019) Italy	Experimental (in vitro)	DPSCs	Group A (Young): 20–23 years (mean 21) Group B (Middle): 42–45 years (mean 43) Group C (Old): 62–66 years (mean 64)	12	NR	Chronological Aging	SA-β-Gal staining, *P16* expression and telomere length	Proliferation and Differentiation (osteogenic, adipogenic and chondrogenic)	*Aged Group*: ↓proliferation ↓osteogenesis ↓adipogenesis →chondrogenesis
Jia et al. (2025) China	Experimental (in vitro*, *in vivo)	PDLSCs	NR	NR	P5-P20	Serial Passaging	SA-β-gal staining and *P21* expression	Proliferation, Differentiation (osteogenic) and Bone Repair (in vivo)	*Late-Passage* ↓proliferation ↓osteogenesis
Jeoung et al. (2015) Korea	Experimental (in vitro)	UC-MSCs-CB	31–34 years (Mothers)	2	P5-P17	Serial Passaging	SA-β-gal staining, Telomerase activity, Expression of *p53, p21, p27*	Proliferation	*Late-Passage* ↓proliferation
Jin et al. (2016) Korea	Experimental (in vitro)	UC-MSCs-CB	NR	27	P5-P15	Serial Passaging	SA-β-Gal staining, *P16, P21* and *P53* expression	Proliferation and Differentiation (osteogenic and adipogenic)	*Late-Passage* ↓proliferation ↓osteogenesis ↓adipogenesis
Katsube et al. (2008) Japan	Experimental (in vitro)	BM-MSCs	1, 25, and 74 years	3	P3	Chronological Aging	SA-β-gal staining and expression of p*16, p21*, and *MMP1*	Proliferation	*Aged Group:* ↓proliferation
Kim et al. (2020) Korea	Experimental (in vitro*, *in vivo)	UC-MSCs-CB	NR	10	P2-P15	Serial Passaging	SA-β-Gal staining, *P16, P21* and *P53* expression	Proliferation	*Late-Passage* ↓proliferation
Kim et al. (2021) Korea	Experimental (in vitro)	BM-MSCs	NR	1	P5-P15	Serial Passaging	SA-β-gal staining, *P15* and *P16* expression	Proliferation and Differentiation (osteogenic and adipogenic)	*Late-Passage* ↓proliferation ↓osteogenesis →adipogenesis
Kornicka et al. (2015) Poland	Experimental (in vitro)	AD-MSCs	>20 (age range 20–29, mean age 24 ± 1.4 years) >50 (age range 50–60, mean age 57.5±0.7) >60 (age range 60–69, age 67) >70 (age range 70–79, mean age 75 ± 2.8)	28	P1-P3	Chronological Aging	SA-β-Gal staining, *P16* and *P53* expression	Proliferation and Differentiation (osteogenic and adipogenic)	*Aged Group*: ↓proliferation ↓osteogenesis ↓/↑adipogenesis
Legzdina et al. (2016) Latvia	Experimental (in vitro)	AD-MSCs	27–63 years (27, 38, 38, 43, 47, 57, 61, 63)	8	P2-P14	Chronological Aging and Serial Passaging	SA-β-Gal staining and relative telomere length analysis	Proliferation and Differentiation (osteogenic and adipogenic)	*Aged Group and Late-Passage*: ↓proliferation ↓osteogenesis ↓adipogenesis
Li et al. (2025) China	Experimental (in vitro)	DPSCs	NR	NR	P3-P12	Serial Passaging	SA-β-gal staining, *P16* expression ROS levels	Differentiation (osteogenic)	*Late-Passage* ↓osteogenesis
Ma et al. (2019) China	Experimental (in vitro*, *in vivo)	DPSCs^a^ PDLSCs^a^ BM-MSCs^b^ AD-MSCs^b^	16–20 years	5	P3-P6	Serial Passaging and Inflammatory Stimuli – LPS and TNF- α	SA-β-Gal staining, *P16, P21* and *P53* expression	Proliferation, Differentiation (osteogenic) and Bone/Periodontal Repair	*Late-Passage* ↓proliferation →^a^/↓^b^osteogenesis ↓bone/periodontal repair *LPS and TNF- α* ↓ osteogenesis ↓ bone/periodontal repair
Scheers et al. (2013) Belgium	Experimental (in vitro*, *in vivo)	UC-MSCs-WJ	Full-term newborns	14	P1-P21	Serial Passaging	SA-β-Gal staining	Proliferation and Differentiation (osteogenic and adipogenic)	*Late-Passage* ↓proliferation ↓osteogenesis ↓adipogenesis
Stolzing et al. (2008) UK	Experimental (in vitro)	BM-MSCs	Young: 7–18 years Adult: 19–40 years Aged: >40 years	33	P1-P5	Chronological Aging	SA-β-Gal staining, *P21* and *P53* expression	Proliferation and Differentiation (osteogenic, adipogenic and chondrogenic)	*Aged Group*: ↓ proliferation ↓osteogenesis ↓adipogenesis ↓chondrogenesis
Truong, et al. (2019) Vietnam	Experimental (in vitro)	AD-MSCs	NR	3	P5-P15	Serial Passaging	SA-β-gal staining	Differentiation (osteogenic, adipogenic and chondrogenic)	*Late-Passage* →osteogenesis →adipogenesis →chondrogenesis
Wagner et al. (2008) Germany	Experimental (in vitro)	BM-MSCs	8, 23, 25, 28, 29, 31, 32, 59 years	8	P2-P12	Chronological Aging and Serial Passaging	SA-β-gal staining	Proliferation and Differentiation (osteogenic and adipogenic)	*Aged Group and Late-Passage*: ↓proliferation ↑osteogenesis ↓adipogenesis
Wang et al. (2025a, b) China	Experimental (in vitro*, *in vivo)	AD-MSCs	NR	NR	P4-P10	Serial Passaging	SA-β-Gal staining, *P16* and *P21* expression	Differentiation (osteogenic, adipogenic and chondrogenic)	*Late-Passage*: ↑osteogenesis ↓adipogenesis ↓chondrogenesis
Wang et al. (2025a) China	Experimental (in vitro)	DPSCs	16–70 years	NR	P3-P12	Chronological Aging and Serial Passaging	SA‑β‑gal staining, γH2AX immunofluorescence, *P16/P21* expression	Proliferation and Differentiation (osteogenic)	*Aged Group and Late-Passage*: ↓proliferation ↓osteogenesis
Wenjing et al. (2024) China	Experimental (in vitro)	BM-MSCs	NR	NR	P5-P15	Serial Passaging	SA-β-gal staining, *p16/p21/p53* expression	Proliferation, Differentiation (osteogenic, adipogenic and chondrogenic) and Migration	*Late-Passage*: ↓proliferation ↓osteogenesis ↓adipogenesis ↓chondrogenesis ↓migration
Wu et al. (2013) Taiwan	Experimental (in vitro)	AD-MSCs	Infant: <1 year (3 mo, 4 mo, 6 mo, 1 yr) Adult: 20–54 years (15, 27, 35, 37, 44, 45) Elderly: >55 years (51, 55, 71)	13	P0-P1	Chronological Aging	Telomere length	Differentiation (osteogenic)	*Aged Group*: ↓osteogenesis
Wu et al. (2022) Taiwan	Experimental (in vitro)	AD-MSCs	NR	6	P3-P10	Chronological Aging and Serial Passaging	SA-β-gal staining, *p16/p21/p53* expression	Proliferation and Differentiation (osteogenic, adipogenic and chondrogenic)	*Aged Group and Late-Passage*: ↓proliferation ↑osteogenesis ↓adipogenesis ↓chondrogenesis
Yang et al. (2021) China	Experimental (in vitro*, *in vivo)	PDLSCs	11–16 years	NR	P3-P12	Serial Passaging	SA-β-gal staining, *p16 and p21* expression	Proliferation, Differentiation (osteogenic) and Migration	*Late-Passage*: ↓proliferation ↓osteogenesis ↓migration ↓bone formation
Ye et al. (2016) China	Experimental (in vitro)	AD-MSCs	Group A (Young): 20–38 years (mean 26.33 ± 5.48); Group B (Old): 50–67 years (mean 56.44 ± 5.83)	20	P0-P9	Chronological Aging	SA-β-Gal staining, *P16* and *P53* expression	Proliferation and Differentiation (osteogenic, adipogenic and chondrogenic)	*Aged Group*: ↓osteogenesis ↓adipogenesis ↓chondrogenesis
Yu et al. (2014) Korea	Experimental (in vitro*, *in vivo)	UC-MSCs-CB	NR	3	P5-P20	Serial Passaging and Inflammatory Stimuli—IFN-ɣ and TNF- α	SA-β-gal staining and *p16* expression	Proliferation and Immune Response	*Late-Passage*: ↓proliferation ↓PGE2 ↓COX-2 →TGF-β1 →NO
Zhang et al. (2024) Australia	Experimental (in vitro)	AD-MSCs	NR	NR	P4-P10	Serial Passaging	SA-β-gal staining, *p16/p21/p53* expression	Proliferation and Differentiation (osteogenic)	*Late-Passage*: ↓proliferation ↓osteogenesis
Zhuang et al. (2015) China	Experimental (in vitro)	UC-MSCs-WJ	Full-term deliveries	10	P3-P15	Serial Passaging	SA-β-Gal staining	Proliferation and Immune Response	*Late-Passage*: ↓proliferation ↑HMOX-1 ↑IL-10 ↑iNOS ↑IL-6 ↓IL-1α ↓IL-1β ↓IFN-ɣ

Methodological characteristics and main findings of included studies on senescence in MSC. The table summarizes the experimental designs, cell types, senescence induction and confirmation methods, assessed outcomes, and key results from the selected studies. *DPSCs* dental pulp stem cells, *AD-MSCs* adipose-derived mesenchymal stem cells, *BM-MSCs* bone marrow mesenchymal stem cells, *UC-MSCs*^*1*^ umbilical cord mesenchymal stem cells derived from Wharton's jelly, *UC-MSCs*^*2*^ umbilical cord mesenchymal stem cells derived from cord blood, *PDLSCs* periodontal ligament stem cells, *SA-β-Gal* senescence-associated β-galactosidase, *LPS* lipopolysaccharide, *NR* not reported. The symbols ↓, ↑, and → denote, respectively, a decrease, increase, or no significant change in the assessed parameter in the senescent group compared to controls

### Results of individual studies

**Fig. 3:**
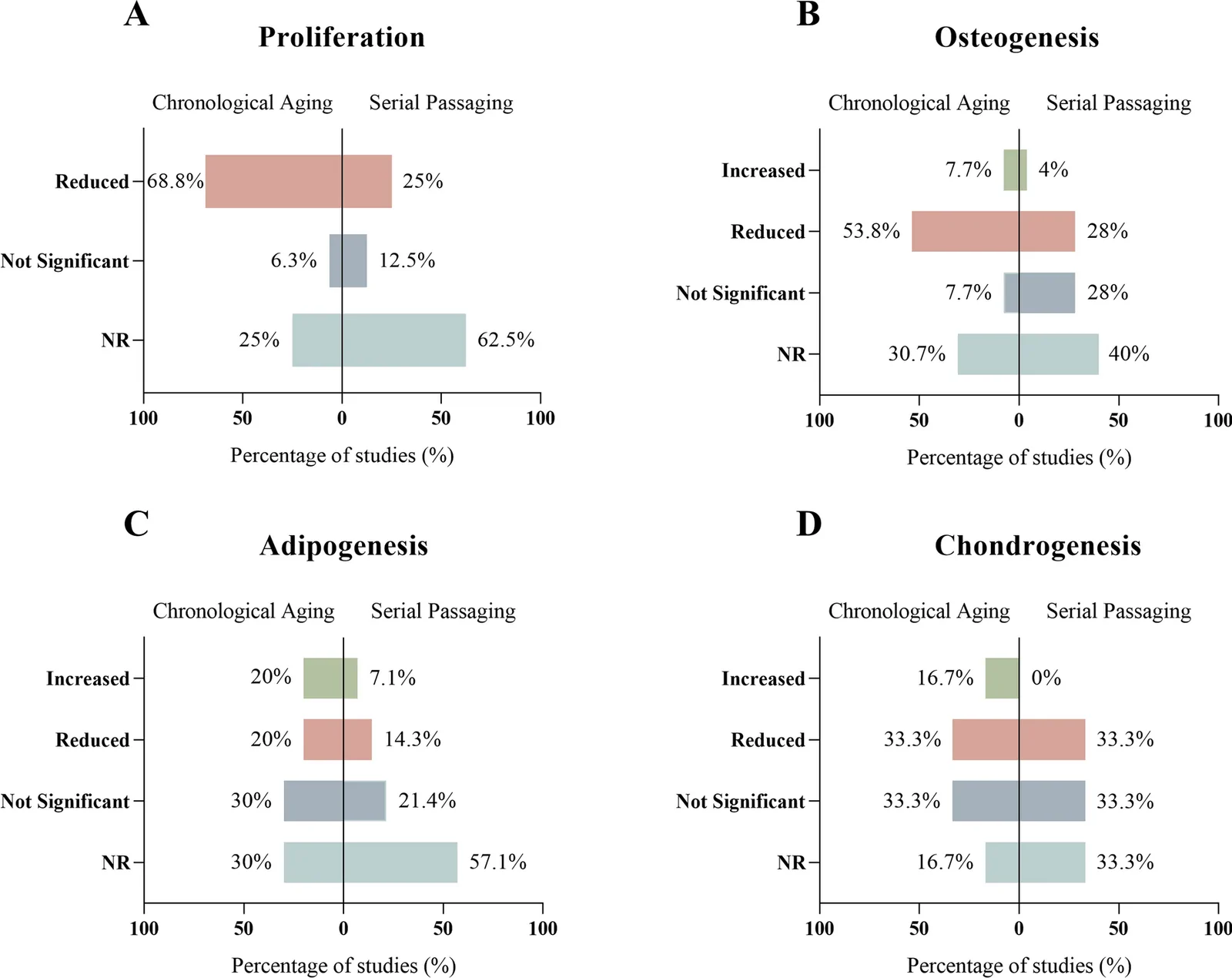
Comparative effects of chronological aging versus serial passaging on MSC functional capacities. Mirrored horizontal bar charts display the percentage of studies reporting increased, reduced, not significant, or not reported outcomes: **A** proliferation, **B** osteogenesis, **C** adipogenesis, and **D** chondrogenesis. Left-side bars represent chronological aging models; right-side bars represent serial passaging models. All values are expressed as percentages of total studies for each functional assay

Results were synthesized according to senescence induction method (chronological aging, serial passaging, or inflammatory stimuli). Due to limited data, immune response, in vivo tissue repair, and inflammatory stimulus–induced senescence were analyzed descriptively. Detailed outcome-specific data are provided in Supplementary Tables 4–10. A graphical summary of proliferation and differentiation outcomes according to chronological aging and serial passaging is presented in Fig. 3.

### Proliferation

Inflammatory stimuli, chronological aging, and serial passaging were frequently associated with reduced proliferative capacity, although this outcome was not uniformly reported across studies (Supplementary Table 3). Repeated inflammatory stimulation led to diminished proliferation in DPSCs (Feng et al. 2014a, 2018). Similarly, chronological aging was predominantly associated with reduced proliferation in AD-MSCs (Gruber et al. 2012; Alt et al. 2012; Choudhery et al. 2014; Kornicka et al. 2015; Wu et al. 2022), BM-MSCs (Huang et al. 2023), and DPSCs (Feng et al. 2014b; Iezzi et al. 2019; Chen et al. 2024; Wang et al. 2025b), although several studies did not perform direct statistical comparisons(Stolzing et al. 2008; Katsube et al. 2008; Wagner et al. 2008) (n = 10, 66.7%). Serial passaging showed a variable but generally negative impact on proliferation (n = 6, 25%), with significant reductions more frequently reported in AD-MSCs (Danisovic et al. 2017; Zhang et al. 2024) and PDLSCs(Hu et al. 2024; Jia et al. 2025), while studies on UC-MSCs rarely performed formal comparative analyses (Christodoulou et al. 2013; Zhuang et al. 2015; Jin et al. 2016; Kim et al. 2021). Studies using serial passaging to induce senescence were included only when independent measures of proliferative capacity were reported, as cumulative population doublings primarily reflect replicative history rather than proliferative function.

### Osteogenic differentiation

Osteogenic differentiation was variably affected by senescence depending on the induction method and cell source (Supplementary Table 4). In a single study evaluating BM-MSCs and DPSCs under inflammatory stimulation, osteogenic differentiation appeared reduced in senescent cells; however, the absence of statistical analysis precludes confirmation of this finding (Ma et al. 2019). Under chronological aging conditions, 7 studies (53.8%) reported reduced osteogenic potential in AD-MSCs (Wu et al. 2013; Choudhery et al. 2014; Kornicka et al. 2015; Ye et al. 2016), BM-MSCs (Stolzing et al. 2008), and DPSCs (Feng et al. 2014b; Iezzi et al. 2019), with occasional reports of preserved (n = 1, 7.7%) or increased (n = 1, 7.7%) differentiation depending on donor characteristics(Horibe et al. 2014; Wu et al. 2022). Serial passaging demonstrated heterogeneous effects: while 7 studies (28%) reported reduced osteogenesis in AD-MSCs(Ma et al. 2019), BM-MSCs (Cheng et al. 2011; Ma et al. 2019), PDLSCs(Yang et al. 2021; Jia et al. 2025), and UC-MSCs (Govarthanan et al. 2025), one study (4%) reported increased osteogenic differentiation in late-passage cells (Cheng et al. 2011), and others found no significant differences (n = 7, 28%), indicating heterogeneous osteogenic outcomes (Gruber et al. 2012; Bertolo et al. 2016; Jin et al. 2016; Diomede et al. 2017; Ma et al. 2019; Wenjing et al. 2024).

### Adipogenic differentiation

The effects of senescence on adipogenic differentiation were variable (Supplementary Table 5). Chronological aging was associated with both increased (Kornicka et al. 2015; Wu et al. 2022) (n = 2, 20%) and reduced (Feng et al. 2014b; Ye et al. 2016) (n = 2, 20%) adipogenic potential in AD-MSCs, whereas BM-MSCs and DPSCs generally showed non-significant (Stolzing et al. 2008; Horibe et al. 2014) (n = 3, 30%) or no reported changes (Iezzi et al. 2019) (n = 3, 30%). Following serial passaging, qualitative or non-comparative analyses predominated, with 8 studies (57.1%) not reporting direct statistical comparisons (Bonab et al. 2006; Wagner et al. 2008; Scheers et al. 2013; Legzdina et al. 2016; Jin et al. 2016; Truong et al. 2019; Kim et al. 2021; Wang et al. 2025a). Among studies providing comparative data, one study (14.3%) reported reduced adipogenic differentiation in senescent cells, with this finding observed in two different MSC populations analyzed within the same study [39]. In contrast, another single study (7.1%) described increased adipogenic differentiation in senescent cells [42].

### Chondrogenic differentiation

Few studies evaluated chondrogenic differentiation of senescent MSCs (Supplementary Table 6). Chronological aging was generally associated with reduced chondrogenic capacity (Choudhery et al. 2014; Ye et al. 2016) (n = 2, 33.3%), with occasional reports of preserved (Stolzing et al. 2008; Iezzi et al. 2019) (n = 2, 33.3%) or enhanced (Wu et al. 2022) (n = 1, 16.7%) differentiation. Serial passaging more consistently impaired chondrogenic differentiation in AD-MSCs (Truong et al. 2019; Wang et al. 2025a), BM-MSCs (Bertolo et al. 2016), and UC-MSCs (Govarthanan et al. 2025), although the limited number of studies precludes firm conclusions.

### Migration

Cell migration was rarely assessed (Supplementary Table 7). However, the available evidence suggests that both chronological aging and serial passaging reduce migratory capacity in DPSCs (Chen et al. 2024), BM-MSCs (Wenjing et al. 2024), and PDLSCs (Yang et al. 2021) (n = 3, 100%).

### Immune response

Data on immunomodulatory function were scarce (Supplementary Table 8). Chronological aging was associated with a phenotypic shift towards a pro-inflammatory profile in BM-MSCs (Huang et al. 2023), although direct comparisons were limited. Serial passaging altered immune-related marker expression in UC-MSCs, generally favoring increased expression of immunosuppressive mediators, such as PGE2, although comparative statistical analyses were not consistently performed (Yu et al. 2014).

### In vivo tissue repair

Evidence regarding in vivo tissue repair capacity was limited (Supplementary Table 9). Inflammation-induced, aging-related, and serial passaging–induced senescence was generally associated with impaired tissue regeneration properties across different MSC sources; however, most studies relied on qualitative or non-comparative analyses (Horibe et al. 2014; Ma et al. 2019; Yang et al. 2021; Chen et al. 2024) (n =4, 80%).

### Additional narrative evidence

One study assessing replicative senescence was not included in the summary tables because cells were grouped according to high versus low proliferative potential rather than donor age, passage number, or inflammatory exposure. Results from this study were therefore synthesized only in narrative form. High-proliferative clones sustained more than 80 population doublings. Osteogenic differentiation, assessed by Alizarin Red staining, was positive at 30 PDs but markedly reduced at 75 PDs, whereas adipogenic differentiation, evaluated by Oil Red O staining, was positive at 30 PDs and increased at 75 PDs. All differentiation outcomes were evaluated qualitatively, without statistical analysis (Alraies et al. 2017).

### Risk of bias in studies

Most in vitro studies demonstrated low overall risk of bias according to the PETRICCS-based assessment, with high reporting adequacy and no studies classified as high risk (Supplementary Fig. 1). Common limitations included insufficient reporting of blinding, randomization, and replicate numbers. In vivo studies assessed using SYRCLE’s risk of bias tool demonstrated low to moderate methodological quality. Frequent deficiencies were observed in domains related to blinding of caregivers/investigators and outcome assessors, random housing procedures, and selective outcome reporting, which may compromise internal validity and limit translational relevance (Supplementary Fig. 2).

## Discussion

Cellular senescence is a fundamental biological process induced by chronological and biological aging, as well as diverse cellular stressors. Beyond its physiological role in development and tissue homeostasis, senescence has emerged as a key driver of organismal aging and age-related diseases, a promising target for therapeutic strategies aimed at modulating aging and aging-associated conditions, and a potential determinant of cell therapy product quality (Kirkland and Tchkonia 2017). Accumulating evidence indicates that cellular senescence is associated with a set of well-defined molecular and functional events; however, investigations across different cell types suggest the existence of cell-type–specific features and responses.

The synthesis of the current literature reveals that the most consistent hallmark of MSC senescence is a marked reduction in proliferative capacity. This decline was observed across all tissue sources and was independent of the induction model employed (chronological aging, serial passaging, or inflammatory stress). This universal loss of proliferation capacity confirms that the core machinery of senescence—primarily the activation of p16 and p21 pathways—is robustly conserved in MSCs (Gruber et al. 2012; Ma et al. 2019). In contrast, the impact of senescence on other functional properties, such as differentiation and immunomodulation, appears far more heterogeneous.

Osteogenic differentiation shows heterogeneous responses, largely dependent on cellular origin and experimental context. While many studies report impaired osteogenesis, others describe preserved or even enhanced osteodifferentiation, particularly in DPSCs and UC-MSCs (Bertolo et al. 2016; Diomede et al. 2017; Ma et al. 2019). Similarly, adipogenic and chondrogenic differentiation outcomes range from reduced to increased potential, with no consistent pattern across senescence models or cell types, with findings ranging from functional impairment to unexpected gain-of-function (Kornicka et al. 2015; Ye et al. 2016). This lack of consistency suggests that the multilineage differentiation program may be more resilient to senescence-associated changes than the cell cycle machinery. The predominance of non-significant findings suggests that differentiation capacity may be less sensitive to senescence than proliferation (Stolzing et al. 2008; Horibe et al. 2014; Diomede et al. 2017; Wenjing et al. 2024). Furthermore, age-group definitions significantly influence outcomes; for instance, studies labeling 'mature adult' donors as senescent rather than focusing on truly elderly populations may mask the full extent of age-related functional decline (Wu et al. 2022).

Although fewer studies evaluated migration, available evidence consistently indicates impaired migratory capacity in senescent MSCs derived from DPSCs, BM-MSCs, and PDLSCs, using both chronological aging and serial passaging models (Chen et al. 2024; Wenjing et al. 2024; Yang et al. 2021). This convergence suggests that migration is a senescence-sensitive function, with potential implications for tissue homing and regenerative efficacy in cell-based therapies and wound repair (Akram et al. 2013). In parallel, senescence is associated with marked alterations in the immunomodulatory profile of MSCs, characterized by increased expression or secretion of pro-inflammatory cytokines such as TNF-α, IL-6, IL-1β, and IL-8, alongside variable regulation of anti-inflammatory mediators including IL-10, IDO-1, and TGF-β1 (Horibe et al. 2014; Yu et al. 2014; Zhuang et al. 2015; Huang et al. 2023). These findings support the concept that senescence shifts the MSC secretome towards SASP, modifying immune regulatory functions rather than uniformly suppressing them (Coppé et al. 2008; Rodier et al. 2009).

Evidence from in vivo studies, although limited, consistently indicates reduced regenerative capacity of senescent MSCs. Across different senescence models, MSCs derived from bone marrow, dental pulp, and periodontal ligament demonstrated impaired mineralization, reduced bone formation, and compromised neo-angiogenesis in regenerative settings (Horibe et al. 2014; Ma et al. 2019; Yang et al. 2021; Chen et al. 2024). In particular, aged/senescent DPSCs exhibited significantly decreased pulp regeneration and vascularized tissue formation compared with young/non-senescent cells, highlighting the functional consequences of senescence in complex tissue environments (Horibe et al. 2014). This compromised reparative potential likely stems not only from the loss of proliferative and differentiation capacity but also from the deleterious effects of the SASP (Lopes-Paciencia et al. 2019; Gnani et al. 2019). In a physiological context, senescent MSCs can actively impair the local microenvironment through a ‘bystander effect,’ where the secretion of pro-inflammatory cytokines and reactive oxygen species propagates senescence to neighboring healthy cells and inhibits the endogenous regenerative response(Acosta et al. 2013). Despite frequent lack of statistical significance, the consistent direction of these findings suggests compromised reparative potential of senescent MSCs in vivo.

Despite these insights, several elements explain the inconsistencies found in the literature and represent limitations of the current evidence. Donor-related factors such as chronological and biological age, genetic background, and underlying inflammatory status contribute to inter-study variability. Methodological differences, including the senescence induction model employed, the level of senescence-induction achieved (i.e. the lack of unified senescence level thresholds), culture conditions, and timing of analysis, might result in divergent molecular and functional outcomes. Furthermore, the lack of standardized criteria for senescence definition—reflected in variability in marker selection and functional readouts—complicates direct comparisons between studies. Such substantial heterogeneity in senescence induction methods, donor age definitions, passages evaluated, and functional assays preclude quantitative synthesis. Small sample sizes and also limited direct statistical comparisons further constrain interpretation. Two studies from the same research group reported highly similar proliferation data (Feng et al. 2014a, 2018), which may limit independence for this outcome. Furthermore, our search strategy may have introduced a selection bias. Although the term “mesenchymal stromal cell” is currently recommended by the International Society for Cell & Gene Therapy (ISCT) (Viswanathan et al. 2019), the search strategy relied on the historically predominant term “mesenchymal stem cell”. Consequently, studies that exclusively used the term “stromal” without referencing “stem” may have been missed, which represents a limitation of this review.

Although some of these factors are inherent to primary cell culture research, collectively, they likely underlie the diverse and sometimes conflicting results reported in the literature regarding the impact of senescence on MSC biology and therapeutic potential. Furthermore, given that most included studies were based on in vitro experiments, with few investigations involving in vivo models and no clinical trials being included, a moderate to high risk of bias in several in vivo studies suggests that the translational interpretation of the findings is indeed limited at this point. Due to these limitations, results were synthesized narratively, and some data extracted from figures may introduce minor imprecision. Overall, the consistent functional impairment observed in senescent MSCs contributes to the understanding of how these cells act throughout aging and underscores donor age and passage number as critical quality parameters in cell manufacturing (Danisovic et al. 2017; Truong et al. 2019).

Senescence-associated changes in inflammatory and immunomodulatory profiles further emphasize the need for careful control of inflammatory stimuli during cell expansion, especially for therapeutic application purposes (Feng et al. 2014a, 2018; Ma et al. 2019). These findings support the implementation of standardized senescence screening, inflammatory profiling, functional quality control criteria, and upper passage limits for clinical-grade MSCs to ensure therapeutic efficacy and reproducibility (Yang et al. 2021; Chen et al. 2024). Future studies should prioritize standardized senescence models, direct comparisons across MSC tissue sources, and greater use of in vivo and translational models with clinically relevant endpoints, as well as strategies to prevent or mitigate senescence-related functional decline.

## Conclusion

This systematic review based on pre-clinical studies demonstrates that cellular senescence consistently affects key MSC properties essential for wound repair and tissue regeneration. Proliferative and migratory capacities are most consistently impaired, while differentiation and immunomodulatory functions vary according to cell source and senescence model. Available in vivo evidence indicates reduced regenerative efficacy of senescent MSCs, contributing to a better understanding of how aged MSCs might contribute to organismal aging. Despite limitations related to methodological heterogeneity and the predominance of in vitro studies, these findings identify donor age, culture expansion, and inflammatory exposure as critical determinants of MSC functional quality. Standardized senescence screening and thresholds, functional quality control measures, and defined passage limits are therefore essential to improving the safety, efficacy, and reproducibility of MSC-based regenerative therapies.

## Supplementary Information

Below is the link to the electronic supplementary material.

Supplementary file1 (DOCX 893 KB)
