# Supplementary material for: Mesenchymal stem/stromal cell senescence in aging and regenerative medicine: a systematic review
Source: Biogerontology. 2026 Sep 25;27(5):167. doi: 10.1007/s10522-026-10501-5 (PMC13615060; doi:10.1007/s10522-026-10501-5)
Supplement: Supplementary file 1 [file 10522_2026_10501_MOESM1_ESM.docx]

**SUPPLEMENTARY TABLE 1** Search Strategy

| **SEARCH** | **QUERY** | **ITEMS FOUND** |
| --- | --- | --- |
| **PUBMED** | ("Stem Cells"[MeSH Terms] OR "Stem Cells"[All Fields] OR "Stem Cell"[All Fields] OR "Mother Cell"[All Fields] OR "Progenitor Cell"[All Fields]) AND ("Cellular Senescence"[MeSH Terms] OR "Cellular Senescence"[All Fields] OR "Cell Senescence"[All Fields] OR "Cell Aging"[All Fields] OR "Cellular Aging"[All Fields] OR "Cell Ageing"[All Fields] OR "Cellular Ageing"[All Fields]) AND ("Regenerative Medicine"[MeSH Terms] OR "Regenerative Medicine"[All Fields] OR "Tissue Engineering"[MeSH Terms] OR "Tissue Engineering"[All Fields] OR "Cell Engineering"[MeSH Terms] OR "Cell Engineering"[All Fields] OR "cell and tissue based therapy"[MeSH Terms] OR "cell and tissue based therapy"[All Fields] OR "Regeneration"[MeSH Terms] OR "Regeneration"[All Fields] OR "Wound Healing"[MeSH Terms] OR "Wound Healing"[All Fields] OR "Wound Repair"[All Fields] OR "Tissue Therapy"[All Fields]) | 2,434 |
| **EMBASE** | ('stem cells'/exp OR 'stem cells' OR 'stem cell'/exp OR 'stem cell' OR 'mother cell'/exp OR 'mother cell' OR 'progenitor cell'/exp OR 'progenitor cell') AND ('cellular senescence' OR 'cell senescence' OR 'cell aging' OR 'cellular aging' OR 'cell ageing' OR 'cellular ageing') AND ('regenerative medicine' OR 'tissue engineering' OR 'cell engineering' OR 'cell- and tissue-based therapy' OR 'regeneration' OR 'wound healing' OR 'wound repair' OR 'tissue therapy') AND ([embase]/lim NOT ([embase]/lim AND [medline]/lim) OR [preprint]/lim) | 968 |
| **COCHRANE** | “Stem Cells” OR “Stem Cell” OR “Mother Cell” OR “Progenitor Cell” in Title Abstract Keyword AND “Cellular Senescence” OR “Cell Senescence” OR “Cell Aging” OR “Cellular Aging” OR “Cell Ageing” OR “Cellular Ageing” in Title Abstract Keyword AND “Regenerative Medicine” OR "Tissue Engineering" OR "Cell Engineering" OR "Cell- and Tissue-Based Therapy" OR "Regeneration" OR "Wound Healing" OR “Wound Repair” OR “Tissue Therapy” in Title Abstract Keyword - (Word variations have been searched) | 9 |
| **WEB OF SCIENCE** | ((ALL=(“Stem Cells” OR “Stem Cell” OR “Mother Cell” OR “Progenitor Cell”)) AND ALL=(“Cellular Senescence” OR “Cell Senescence” OR “Cell Aging” OR “Cellular Aging” OR “Cell Ageing” OR “Cellular Ageing”)) AND ALL=(“Regenerative Medicine” OR “Tissue Engineering” OR "Cell Engineering" OR "Cell- and Tissue-Based Therapy" OR "Regeneration" OR "Wound Healing" OR “Wound Repair” OR “Tissue Therapy”) | 1,149 |
| **GOOGLE SCHOLAR** | "stem cell" AND "cell senescence" AND "regeneration" | 100 |
| **PROQUEST** | (“Stem Cells” OR “Stem Cell” OR “Mother Cell” OR “Progenitor Cell”) AND (“Cellular Senescence” OR “Cell Senescence” OR “Cell Aging” OR “Cellular Aging” OR “Cell Ageing” OR “Cellular Ageing”) AND (“Regenerative Medicine” OR "Tissue Engineering" OR "Cell Engineering" OR "Cell- and Tissue-Based Therapy" OR "Regeneration" OR "Wound Healing" OR “Wound Repair” OR “Tissue Therapy”) | 197 |

**Supplementary Table 1.** Detailed search strategies used in each database. The initial search was conducted on 28 February 2025 and updated on 12 December 2025; all databases were searched on the same day for each search.

**SUPPLEMENTARY TABLE 2** Excluded articles and reasons for exclusion (n=80).

| Reference | Reason for exclusion |
| --- | --- |
| Flores I, Blasco MA. A p53-dependent response limits epidermal stem cell functionality and organismal size in mice with short telomeres. PLoS One. 2009;4(3):e4934. doi: 10.1371/journal.pone.0004934. Epub 2009 Mar 19. PMID: 19295915; PMCID: PMC2654505. | 1 |
| Madonna R., De Caterina R., Willerson J.T., Geng Y.-J. (2011). Adipose tissue-derived mesenchymal stem cells with myocardin-a and telomerase co-expression possess augmented capacity of proliferation and promyogenic activities, American Heart Association's Scientific Sessions 2011. Circulation. | 8 |
| Scheers I., Paganelli M., Lombard C., Najimi M., Sokal E.M. (2011). Umbilical cord matrix stem cells maintain a stable genotype after long-term in vitro culture, 62nd Annual Meeting of the American Association for the Study of Liver Diseases: The Liver Meeting 2011. Hepatology. http://dx.doi.org/10.1002/hep.24666 | 8 |
| Goichberg P., Kannappan R., Bai Y., Fiorini C., D'Amario D., Sanada F., Pesapane A., Arranto C., Kim J., Anastasia L., Gurusamy N., D'Alessandro D.A., Michler R.E., Zheng H., Hosoda T., Rota M., Kajstura J., Bolli R., Anversa P., Leri A. (2011). Alterations in ephrin A1/EphA2 signaling causes defects in the migration of senescent human cardiac stem cells, American Heart Association's Scientific Sessions 2011. Circulation. | 8 |
| Scheers I., Maerckx C., Decottignies A., Paganelli M., Najimi M., Sokal E. (2011). Long-term in vitro cultured umbilical cord matrix stemcells maintain a stable genotype after long-term in vitro culture, European Society for Paediatric Gastroenterology, Hepatology, and Nutrition Annual Meeting 2011. Journal of Pediatric Gastroenterology and Nutrition. http://dx.doi.org/10.1097/MPG.0b013e318224e326 | 8 |
| Klotz B, Mentrup B, Regensburger M, Zeck S, Schneidereit J, Schupp N, Linden C, Merz C, Ebert R, Jakob F. 1,25-dihydroxyvitamin D3 treatment delays cellular aging in human mesenchymal stem cells while maintaining their multipotent capacity. PLoS One. 2012;7(1):e29959. doi: 10.1371/journal.pone.0029959. Epub 2012 Jan 5. PMID: 22242193; PMCID: PMC3252365. | 4 |
| Lapasset L., Milhavet O., Pellestor F., Prieur A., Aït-Hamou N., Lagnado A., Schwerer H., Besnard E., Babled A., Ramirez J.M., De Vos J., Lehmann S., Lemaitre J.-M. (2013). Exploring strategies for cell rejuvenation and tissues regeneration, 23rd Annual Meeting of the European Tissue Repair Society. Wound Repair and Regeneration. | 8 |
| Chen C., Sun K., Geng S., Liu J., Zhou Y., Wang L., Wang J.-W., Huang G.-N., Wang Y.-P. (2013). Effect of ginsenoside Rg1 on radiation-induced senescence of hematopoietic stem/progenitor cells and relevant mechanism. Chinese Journal of Biologicals, 26(11), 1604-1609+1616. | 7 |
| Cipriani P, Di Benedetto P, Liakouli V, Del Papa B, Di Padova M, Di Ianni M, Marrelli A, Alesse E, Giacomelli R. Mesenchymal stem cells (MSCs) from scleroderma patients (SSc) preserve their immunomodulatory properties although senescent and normally induce T regulatory cells (Tregs) with a functional phenotype: implications for cellular-based therapy. Clin Exp Immunol. 2013 Aug;173(2):195-206. doi: 10.1111/cei.12111. PMID: 23607751; PMCID: PMC3722920. | 2 |
| Gullo F, De Bari C. Prospective purification of a subpopulation of human synovial mesenchymal stem cells with enhanced chondro-osteogenic potency. Rheumatology (Oxford). 2013 Oct;52(10):1758-68. doi: 10.1093/rheumatology/ket205. Epub 2013 Jun 26. PMID: 23804221. | 4 |
| Jun HS, Dao LT, Pyun JC, Cho S. Effect of cell senescence on the impedance measurement of adipose tissue-derived stem cells. Enzyme Microb Technol. 2013 Oct 10;53(5):302-6. doi: 10.1016/j.enzmictec.2013.07.001. Epub 2013 Jul 16. PMID: 24034428. | 5 |
| He X., Li X., Bao H.-J., Wang R.-F., Liu Y.-D., Song S.-W. (2014). Effects of three-dimensional spheroid culture system on biological characteristics of mouse bone marrow mesenchymal stem cells. Chinese Journal of Tissue Engineering Research, 18(45), 7227-7232. http://dx.doi.org/10.3969/j.issn.2095-4344.2014.45.003 | 7 |
| Klinkhammer BM, Kramann R, Mallau M, Makowska A, van Roeyen CR, Rong S, Buecher EB, Boor P, Kovacova K, Zok S, Denecke B, Stuettgen E, Otten S, Floege J, Kunter U. Mesenchymal stem cells from rats with chronic kidney disease exhibit premature senescence and loss of regenerative potential. PLoS One. 2014 Mar 25;9(3):e92115. doi: 10.1371/journal.pone.0092115. PMID: 24667162; PMCID: PMC3965415. | 2 |
| Kumar D., Rooge S.B., Shubham S., Anand L., Mohanty S., Bihari C., Kumar A., Sarin S.K. (2015). Bone marrow mesenchymal stem cells (BMSCS) from decompensated cirrhotics show premature senescence and limited tissue repair and regenerative potential, 66th Annual Meeting of the American Association for the Study of Liver Diseases: The Liver Meeting 2015. Hepatology. http://dx.doi.org/10.1002/hep.28216 | 2 |
| Machado AK, Cadoná FC, Azzolin VF, Dornelles EB, Barbisan F, Ribeiro EE, *et al*. Guaraná (Paulinia cupana) improves the proliferation and oxidative metabolism of senescent adipocyte stem cells derived from human lipoaspirates. Food Res Int. 2015;67:426-33. | 5 |
| Cho JG, Lee JH, Hong SH, Lee HN, Kim CM, Kim SY, Yoon KJ, Oh BJ, Kim JH, Jung SY, Asahara T, Kwon SM, Park SG. Tauroursodeoxycholic acid, a bile acid, promotes blood vessel repair by recruiting vasculogenic progenitor cells. Stem Cells. 2015 Mar;33(3):792-805. doi: 10.1002/stem.1901. PMID: 25407160. | 1 |
| Windmolders S, Willems L, Daniëls A, Linsen L, Fanton Y, Hendrikx M, Koninckx R, Rummens JL, Hensen K. Clinical-scale in vitro expansion preserves biological characteristics of cardiac atrial appendage stem cells. Cell Prolif. 2015 Apr;48(2):175-86. doi: 10.1111/cpr.12166. Epub 2015 Jan 29. PMID: 25630660; PMCID: PMC6495491. | 4 |
| Li Y., Zhou Y., Wang Y.-P., Wang J.-W., He Y.-H., Ding J.-C., Zhao W.-Z., Yang Y.-Q., Wang H.-J. (2016). Effect of SIRT1/NF-κB signal axis on delaying hematopoietic stem cell and progenitor cell senescence with ginsenoside R1 in aging model rat induced by D-galactose. Chinese Traditional and Herbal Drugs, 47(22), 4016-4020. http://dx.doi.org/10.7501/j.issn.0253-2670.2016.22.014 | 7 |
| Tsai T.-L., Li W.-J. (2016). Soluble factors in bone marrow niche enhance human mesenchymal stem cell-based tissue regeneration, 2016 Annual Meeting of the Orthopaedic Research Society. Journal of Orthopaedic Research. http://dx.doi.org/10.1002/jor.23247 | 8 |
| Marycz K, Tomaszewski KA, Kornicka K, Henry BM, Wroński S, Tarasiuk J, Maredziak M. Metformin Decreases Reactive Oxygen Species, Enhances Osteogenic Properties of Adipose-Derived Multipotent Mesenchymal Stem Cells In Vitro, and Increases Bone Density In Vivo. Oxid Med Cell Longev. 2016;2016:9785890. doi: 10.1155/2016/9785890. Epub 2016 Apr 18. Erratum in: Oxid Med Cell Longev. 2017;2017:5923818. doi: 10.1155/2017/5923818. PMID: 27195075; PMCID: PMC4852347. | 4 |
| Tsai T., Li W. (2016). Bone marrow-extracted soluble factors maintain properties of human mesenchymal stem cells, 2016 TERMIS - Americas Conference. Tissue Engineering - Part A. http://dx.doi.org/10.1089/ten.tea.2016.5000.abstracts | 8 |
| Lee J, Byeon JS, Lee KS, Gu NY, Lee GB, Kim HR, Cho IS, Cha SH. Chondrogenic potential and anti-senescence effect of hypoxia on canine adipose mesenchymal stem cells. Vet Res Commun. 2016 Mar;40(1):1-10. doi: 10.1007/s11259-015-9647-0. Epub 2015 Dec 10. PMID: 26661466. | 5 |
| Maciel R.C., Jiang J., Leong N.L., Yamaguchi K.T., Kabir N., Petrigliano F.A., McAllister D.R. (2017). Adipose derived perivascular stem cells are a superior cell source for ligament tissue engineering, 2017 Annual Meeting of the Orthopaedic Research Society. Journal of Orthopaedic Research. | 8 |
| Gilpin S.E., Li Q., Evangelista-Leite D., Frey B.L., Ott H.C. (2017). Enhanced epithelial regeneration on human neonatal lung derived scaffolds is mediated by tenascin-C, American Thoracic Society International Conference, ATS 2017. American Journal of Respiratory and Critical Care Medicine. http://dx.doi.org/10.1164/ajrccm-conference.2017.A19 | 8 |
| OConnor K., Madsen S., Russell K., Tucker A., Glowacki J., Bunnell B. (2017). CD264: A predictor of MSC regenerative potential, Tissue Engineering and Regenerative Medicine International Society's - Americas Annual Conference, TERMIS 2017. Tissue Engineering - Part A. http://dx.doi.org/10.1089/ten.tea.2017.29003.abstracts | 8 |
| Rasmus M., Maj M., Balcerczyk D., Buhl M., Warda K., Jundzill A., Buchholz L., Kloskowski T., Pokrywczynska M., Drewa T. (2017). Adipose derived stem cell properties in long-term culture-an implications for regenerative medicine, 4th Baltic Meeting in Conjunction with the EAU. European Urology, Supplements. http://dx.doi.org/10.1016/S1569-9056(17)31372-6 | 8 |
| Tsai TL, Li WJ. Identification of Bone Marrow-Derived Soluble Factors Regulating Human Mesenchymal Stem Cells for Bone Regeneration. Stem Cell Reports. 2017 Feb 14;8(2):387-400. doi: 10.1016/j.stemcr.2017.01.004. Epub 2017 Feb 2. PMID: 28162996; PMCID: PMC5312426. | 4 |
| Mobarak H, Fathi E, Farahzadi R, Zarghami N, Javanmardi S. L-carnitine significantly decreased aging of rat adipose tissue-derived mesenchymal stem cells. Vet Res Commun. 2017 Mar;41(1):41-47. doi: 10.1007/s11259-016-9670-9. Epub 2016 Dec 10. PMID: 27943151. | 4 |
| Jiang T, Xu G, Wang Q, Yang L, Zheng L, Zhao J, Zhang X. In vitro expansion impaired the stemness of early passage mesenchymal stem cells for treatment of cartilage defects. Cell Death Dis. 2017 Jun 1;8(6):e2851. doi: 10.1038/cddis.2017.215. Erratum in: Cell Death Dis. 2019 Sep 26;10(10):716. doi: 10.1038/s41419-019-1939-9. PMID: 28569773; PMCID: PMC5520885. | 4 |
| Sanap A, Chandravanshi B, Shah T, Tillu G, Dhanushkodi A, Bhonde R, Joshi K. Herbal pre-conditioning induces proliferation and delays senescence in Wharton's Jelly Mesenchymal Stem Cells. Biomed Pharmacother. 2017 Sep;93:772-778. doi: 10.1016/j.biopha.2017.06.107. Epub 2017 Jul 15. PMID: 28724259. | 4 |
| Gilpin SE, Li Q, Evangelista-Leite D, Ren X, Reinhardt DP, Frey BL, Ott HC. Fibrillin-2 and Tenascin-C bridge the age gap in lung epithelial regeneration. Biomaterials. 2017 Sep;140:212-219. doi: 10.1016/j.biomaterials.2017.06.027. Epub 2017 Jun 22. PMID: 28662401; PMCID: PMC5553709. | 4 |
| Colasuonno F, Borghi R, Niceforo A, Muzzi M, Bertini E, Di Giulio A, Moreno S, Compagnucci C. Senescence-associated ultrastructural features of long-term cultures of induced pluripotent stem cells (iPSCs). Aging (Albany NY). 2017 Oct 23;9(10):2209-2222. doi: 10.18632/aging.101309. PMID: 29064821; PMCID: PMC5680563. | 6 |
| Gao S, Xiang C, Qin K, Sun C. Mathematical Modeling Reveals the Role of Hypoxia in the Promotion of Human Mesenchymal Stem Cell Long-Term Expansion. Stem Cells Int. 2018 May 14;2018:9283432. doi: 10.1155/2018/9283432. PMID: 29861746; PMCID: PMC5976908. | 6 |
| Conley S.M., Hickson L.J., Tang H., Jordan K.L., Abumoawad A.M., Kim S.R., Zhu X.-Y., Tchkonia T., Kirkland J.L., Lerman L.O. (2019). Obesity induces mesenchymal stem cell senescence and dysfunction, Midwest Clinical and Translational Research Meeting 2019. Journal of Investigative Medicine. http://dx.doi.org/10.1136/jim-2019-midwestern2019.98 | 2 |
| Chen B, Sun Y, Zhang J, Zhu Q, Yang Y, Niu X, Deng Z, Li Q, Wang Y. Human embryonic stem cell-derived exosomes promote pressure ulcer healing in aged mice by rejuvenating senescent endothelial cells. Stem Cell Res Ther. 2019 May 21;10(1):142. doi: 10.1186/s13287-019-1253-6. PMID: 31113469; PMCID: PMC6528288. | 1 |
| Cui H, Tang D, Garside GB, Zeng T, Wang Y, Tao Z, Zhang L, Tao S. Wnt Signaling Mediates the Aging-Induced Differentiation Impairment of Intestinal Stem Cells. Stem Cell Rev Rep. 2019 Jun;15(3):448-455. doi: 10.1007/s12015-019-09880-9. PMID: 30790135; PMCID: PMC6534527. | 5 |
| Theys N., Episkopou H., Pierard C., Decottignies A. (2020). Assessment of senescence in adipose mesenchymal stem cells for optimized ex vivo expansion and therapeutic potential, ECTS Congress 2020. Bone Reports. http://dx.doi.org/10.1016/j.bonr.2020.100367 | 8 |
| Ghannoum D., Roeder E., El Ouafy M., Cauchois G., Charif N., de Isla N. (2020). Evaluation of fetal mesenchymal stromal/stem cells senescence during in vitro amplification for therapeutic purpose: choice of cell quality parameters, ISCT 2020 Annual Meeting. Cytotherapy. http://dx.doi.org/10.1016/j.jcyt.2020.03.141 | 8 |
| Hu W, Zhu S, Fanai ML, Wang J, Cai J, Feng J. 3D co-culture model of endothelial colony-forming cells (ECFCs) reverses late passage adipose-derived stem cell senescence for wound healing. Stem Cell Res Ther. 2020 Aug 14;11(1):355. doi: 10.1186/s13287-020-01838-w. PMID: 32795343; PMCID: PMC7427858. | 5 |
| Zhang WT, Zhou Q, He C. Effects of Hydrogen Peroxide on the Senescence of Human Mesenchymal Stem Cells Through Fatty Acid 2-Hydroxylase. J Biomater Tissue Eng. 2020 Nov 1;10(11):1634-8. DOI: 10.1166/jbt.2020.2474 | 7 |
| Hong T, Kim MY, Da Ly D, Park SJ, Eom YW, Park KS, Baik SK. Ca2+-activated mitochondrial biogenesis and functions improve stem cell fate in Rg3-treated human mesenchymal stem cells. Stem Cell Res Ther. 2020 Nov 4;11(1):467. doi: 10.1186/s13287-020-01974-3. PMID: 33148318; PMCID: PMC7640456. | 4 |
| Li X, Ma X, Chen Y, Peng D, Wang H, Chen S, Xiao Y, Li L, Zhou H, Cheng F, Gao Y, Chang J, Cheng T, Liu L. Coinhibition of activated p38 MAPKα and mTORC1 potentiates stemness maintenance of HSCs from SR1-expanded human cord blood CD34+ cells via inhibition of senescence. Stem Cells Transl Med. 2020 Dec;9(12):1604-1616. doi: 10.1002/sctm.20-0129. Epub 2020 Jun 29. PMID: 32602209; PMCID: PMC7695631. | 6 |
| Fu S, Zhang C, Yan X, Li D, Wang Y, Dong C, Cao Z, Ning Y, Shao C, Yang T. Primary Cilia as a Biomarker in Mesenchymal Stem Cells Senescence: Influencing Osteoblastic Differentiation Potency Associated with Hedgehog Signaling Regulation. Stem Cells Int. 2021 Jan 26;2021:8850114. doi: 10.1155/2021/8850114. PMID: 33574852; PMCID: PMC7857927. | 5 |
| Sun Y, Zhang J, Chen B, Yang Y, Li H, Niu X, *et al*. Small extracellular vesicles secreted by urine-derived stem cells enhanced wound healing in aged mice by ameliorating cellular senescence. J Mater Sci Technol. 2021;63:216-27. doi: 10.1016/j.jmst.2020.03.014. | 1 |
| Kim SR, Zou X, Tang H, Puranik AS, Abumoawad AM, Zhu XY, Hickson LJ, Tchkonia T, Textor SC, Kirkland JL, Lerman LO. Increased cellular senescence in the murine and human stenotic kidney: Effect of mesenchymal stem cells. J Cell Physiol. 2021 Feb;236(2):1332-1344. doi: 10.1002/jcp.29940. Epub 2020 Jul 13. PMID: 32657444; PMCID: PMC7900892. | 2 |
| Labusca L, Herea DD, Emanuela Minuti A, Stavila C, Danceanu C, Plamadeala P, Chiriac H, Lupu N. Magnetic Nanoparticles and Magnetic Field Exposure Enhances Chondrogenesis of Human Adipose Derived Mesenchymal Stem Cells But Not of Wharton Jelly Mesenchymal Stem Cells. Front Bioeng Biotechnol. 2021 Oct 18;9:737132. doi: 10.3389/fbioe.2021.737132. PMID: 34733830; PMCID: PMC8558412. | 4 |
| Le HM, Nguyen LT, Hoang DH, Bach TQ, Nguyen HTN, Mai HT, Trinh DP, Nguyen TD, Nguyen LT, Than UTT. Differential Development of Umbilical Cord-Derived Mesenchymal Stem Cells During Long-Term Maintenance in Fetal Bovine Serum-Supplemented Medium and Xeno- and Serum-Free Culture Medium. Cell Reprogram. 2021 Dec;23(6):359-369. doi: 10.1089/cell.2021.0050. Epub 2021 Nov 8. PMID: 34748399. | 7 |
| Chuan T., Xiangqing Z., Zailing Y., Donghai Y., Ye L., Yanying W., Yukun Y., Jie H., Guanke L., Xuemin C., Liping S., Zhixu H., Xinghua P. (2022). Bone marrow mesenchymal stem cells regulate ovarian aging in macaques. Chinese Journal of Tissue Engineering Research, 26(7), 1033-1039. http://dx.doi.org/10.12307/2022.133 | 4 |
| Choi D.H., Oh S.-Y., Lee K.E., Jo I., Park Y.S. (2022). Changes in the integrin a3 expression controls tonsil derived mesenchymal stem cell proliferation and senescence, Tissue Engine International Society Asia Engineering and Regenerative Medicine Asia-Pacific Chapter Conference, TERMIS-AP 2022. Tissue Engineering - Part A. http://dx.doi.org/10.1089/ten.tea.2022.29037.abstract.index | 8 |
| Gao Y., Chi Y., Chen Y., Li H.Y., Wang W., Zheng W., Zhu P., An J., Duan Y., Sun T., Xiaofan L., Xue F., Liu W., Fu R., Han Z., Zhang Y., Yang R., Tao C., Wei J., ... Zhang L. (2022). Multi-Omics Analysis of Human Mesenchymal Stem Cell Guides Perinatal MSC Based Cellular Therapy for Splenectomy-Nonresponsive Refractory Immune Thrombocytopenia: A Phase I Clinical Trial, 64th ASH Annual Meeting. Blood. http://dx.doi.org/10.1182/blood-2022-156764 | 8 |
| Khorraminejad-Shirazi M., Sani M., Talaei-Khozani T., Dorvash M., Mirzaei M., Faghihi M.A., Monabati A., Attar A. (2022). AICAR AND NAM SYNERGISTICALLY ATTENUATE SENESCENCE-ASSOCIATED CHANGES IN MESENCHYMAL STEM CELLS: THE INTERPLAY OF AUTOPHAGY AND MTORC1, 6th Tissue Engineering and Regenerative Medicine International Society's - Americas Annual Conference, TERMIS 2021. Tissue Engineering - Part A. http://dx.doi.org/10.1089/ten.tea.2022.29025.abstracts | 8 |
| Narcisi R., Voskamp C., Koevoet W.J., van Osch G.J. (2022). CELLULAR SENESCENCE IMPAIRS CHONDROGENIC DIFFERENTIATION OF MESENCHYMAL STEM/STROMAL CELLS BY INHIBITING THE TRASFORMING GROWTH FACTOR-BETA SIGNALING, 2022 OARSI World Congress on Osteoarthritis. Osteoarthritis and Cartilage. http://dx.doi.org/10.1016/j.joca.2022.02.570 | 8 |
| Cruciani S, Garroni G, Pala R, Barcessat ARP, Facchin F, Ventura C, Fozza C, Maioli M. Melatonin finely tunes proliferation and senescence in hematopoietic stem cells. Eur J Cell Biol. 2022 Jun-Aug;101(3):151251. doi: 10.1016/j.ejcb.2022.151251. Epub 2022 Jun 22. PMID: 35772322. | 2 |
| Choi D., Lee K., Oh S., Lee S., Hwang J., Jo B., Lee J., Park Y., Park K., Jo I. (2023). Tissue Engineering: TONSIL-DERIVED MESENCHYMAL STEM CELLS INCORPORATED IN REACTIVE OXYGEN SPECIES RELEASING HYDROGEL PROMOTE REJUVENATION AND BONE FORMATION THROUGH INCREASED REGULATION OF CELL SURFACE GRP78, 29th Annual ISCT Meeting. Cytotherapy. http://dx.doi.org/10.1016/S1465-3249(23)00712-0 | 8 |
| Go Y.-Y., Lee C.-M., Chae S.-W., Song J.-J. (2023). Regenerative capacity of trophoblast stem cell-derived extracellular vesicles on mesenchymal stem cells. Biomaterials Research, 27(1). http://dx.doi.org/10.1186/s40824-023-00396-5 | 4 |
| Dadashpour M., Mahmoudi H., Rahimi Z., Janghorbanian Poodeh R., Mousazadeh H., Firouzi-Amandi A., Yazdani Y., Nezami Asl A., Akbarzadeh A. (2023). Sustained in vitro delivery of metformin-loaded mesoporous silica nanoparticles for delayed senescence and stemness preservation of adipose-derived stem cells. Journal of Drug Delivery Science and Technology, 87. http://dx.doi.org/10.1016/j.jddst.2023.104769 | 7 |
| Ishiuchi N, Nakashima A, Maeda S, Miura Y, Miyasako K, Sasaki K, Uchiki T, Sasaki A, Nagamatsu S, Nakao N, Nagao M, Masaki T. Comparison of therapeutic effects of mesenchymal stem cells derived from superficial and deep subcutaneous adipose tissues. Stem Cell Res Ther. 2023 May 4;14(1):121. doi: 10.1186/s13287-023-03350-3. PMID: 37143086; PMCID: PMC10161523. | 4 |
| Elsied MA, Sharawi ZW, Al-Amrah H, Hegazy RA, Mohamed AE, Saleh RM, El-Kholy SS, Farrag FA, Fayed MH, El-Magd MA. Walnut Kernel Oil and Defatted Extracts Enhance Mesenchymal Stem Cell Stemness and Delay Senescence. Molecules. 2023 Aug 28;28(17):6281. doi: 10.3390/molecules28176281. PMID: 37687109; PMCID: PMC10488345. | 4 |
| Hejazi, Sajjad1,2; Maleki, Masoud3; Rasekh, Morteza4. Study of the Cellular Senescence Process in Human Umbilical Cord Wharton's Jelly-derived Mesenchymal Stem Cells. Advances in Human Biology 13(4):p 361-366, Oct–Dec 2023. \| DOI: 10.4103/aihb.aihb_19_23 | 6 |
| Mapping aged stem cell states associated with decline in skeletal muscle regeneration. Nat Aging 4, 1680–1681 (2024). https://doi.org/10.1038/s43587-024-00768-z | 8 |
| Pei YA, Patel J, Pei M. Extracellular Matrix Tunes the Regenerative Potential of Fetal Stem Cells. Applied Sciences. 2024; 14(5):1932. https://doi.org/10.3390/app14051932 | 4 |
| Rajput SN, Naeem BK, Ali A, Salim A, Khan I. Expansion of human umbilical cord derived mesenchymal stem cells in regenerative medicine. World J Stem Cells. 2024 Apr 26;16(4):410-433. doi: 10.4252/wjsc.v16.i4.410. PMID: 38690517; PMCID: PMC11056638. | 4 |
| Sun Y, Yu Y, Ma S, Liao C, Yang J, Lyu Y, Zhang X, Zhang J, Tian W, Liao L. Nanotube topography rejuvenates the senescence of mesenchymal stem cells by activating YAP signalling. J Mater Chem B. 2024 Jul 17;12(28):6917-6926. doi: 10.1039/d3tb02828c. PMID: 38904147. | 7 |
| Li F, Zhang F, Shi H, Xia H, Wei X, Liu S, Wu T, Li Y, Shu F, Chen M, Li J, Duan R. Aerobic exercise suppresses CCN2 secretion from senescent muscle stem cells and boosts muscle regeneration in aged mice. J Cachexia Sarcopenia Muscle. 2024 Oct;15(5):1733-1749. doi: 10.1002/jcsm.13526. Epub 2024 Jun 26. PMID: 38925632; PMCID: PMC11446704. | 5 |
| Kang GH, Shin YK, Lim KM, Kim SJ, Song M, Song K, Kim JH, Kim DY, Shin HC, Shin HJ, Cho SG. Thermostable bFGF Improves Cell Lifespan by Enhancing Cell Activity in the Long-Term Culture of Human Orbicularis Oculi Stem Cells. Int J Stem Cells. 2025 Aug 30;18(3):301-310. doi: 10.15283/ijsc24039. Epub 2024 Oct 23. PMID: 39439110; PMCID: PMC12394077. | 4 |
| Sun H, Zhai H, Han K, Ma H, Tan Y, Li S, Liu Z, Cheng L, Lu Q, Zhou L, Liu P. Clinical outcomes of autologous adipose-derived mesenchymal stem cell combined with high tibial osteotomy for knee osteoarthritis are correlated with stem cell stemness and senescence. J Transl Med. 2024 Nov 18;22(1):1039. doi: 10.1186/s12967-024-05814-3. PMID: 39558365; PMCID: PMC11575038. | 2 |
| Xu L, Ren W, Long Y, Yang B, Chen L, Chen W, Chen S, Cao Y, Wu D, Qu J, Li H, Yu Y, Zhang A, Wang S, Wang H, Chen T, Fan G, Li Q, Chen Z. Antisenescence Expansion of Mesenchymal Stem Cells Using Piezoelectric β-Poly(vinylidene fluoride) Film-Based Culture. ACS Appl Mater Interfaces. 2024 Nov 20;16(46):63207-63224. doi: 10.1021/acsami.4c12725. Epub 2024 Nov 6. PMID: 39503875. | 4 |
| Gu K, Feng XM, Sun SQ, Hao XY, Wen Y. Yes-associated protein-mediated melatonin regulates the function of periodontal ligament stem cells under oxidative stress conditions. World J Stem Cells. 2024 Nov 26;16(11):926-943. doi: 10.4252/wjsc.v16.i11.926. PMID: 39619874; PMCID: PMC11606347. | 4 |
| Liu DY, Wu J, Zhou HY, Lv JX, Cai KZ, Tang CB. Phytic acid improves osteogenesis and inhibits the senescence of human bone marrow mesenchymal stem cells under high-glucose conditions via the ERK pathway. Chem Biol Interact. 2024 Jan 5;387:110818. doi: 10.1016/j.cbi.2023.110818. Epub 2023 Nov 22. PMID: 38000455. | 2 |
| Li XY, Zhang L, Sun Y. [Mitochondrial transfer contributes to the odontogenic differentiation of dental mesenchymal stem cells]. Zhonghua Kou Qiang Yi Xue Za Zhi. 2025 Jan 9;60(1):43-53. Chinese. doi: 10.3760/cma.j.cn112144-20240926-00360. PMID: 39743364. | 4 |
| Kaokaen P, Pangjantuk A, Kunhorm P, Promjantuek W, Chaicharoenaudomrung N, Noisa P. Conditioned medium of human umbilical cord-mesenchymal stem cells cultivated with human cord blood serum enhances stem cell stemness and secretome profiles. Toxicol In Vitro. 2025 Mar;103:105973. doi: 10.1016/j.tiv.2024.105973. Epub 2024 Nov 17. PMID: 39561911. | 4 |
| Habibi Roudkenar H., Bahmani B., Halabian R. (2014). Over expression of neutrophil associated lipocalin in bone marrow derived-mesenchymal stem cells improve their regenerative potential trough reducing of senescent cells, 33rd International Congress of the International Society of Blood Transfusion, in Conjunction with the 33rd Congress of the KSBT and the 2014 Congress of the Korean Hematology Societies. Vox Sanguinis. http://dx.doi.org/10.1111/vox.12155 | 8 |
| Lee H.C., Wong C.Y., Nguyen Phan N.N., Cheong S.K., Choo K.B., Kamarul T. (2013). Establishment and characterization of replicate senescence study models of human mesenchymal stem cells, 19th Annual Meeting of the International Society for Cellular Therapy, ISCT 2013. Cytotherapy. http://dx.doi.org/10.1016/j.jcyt.2013.01.054 | 8 |
| Ohgo S, Hasegawa S, Hasebe Y, Mizutani H, Nakata S, Akamatsu H. Senescent dermal fibroblasts enhance stem cell migration through CCL2/CCR2 axis. Exp Dermatol. 2015 Jul;24(7):552-4. doi: 10.1111/exd.12701. Epub 2015 May 4. PMID: 25808810. | 4 |
| Roudkenar M.H., Roushandeh A.M. (2017). Ngal protects bone marrow-derived mesenchymal stem cells under sub-lethal doses of oxidative stress conditions by regulation of senescence, 2nd National Festival and International Congress on Stem Cell and Regenerative Medicine. Iranian Journal of Biotechnology. http://dx.doi.org/10.15171/ijb.2017.s1-334 | 8 |
| Severino V., Alessio N., Farina A., Galderisi U., Chambery A. (2013). Secretome profiling of senescent mesenchymal stem cells (MSC) by high resolution LC-MS analysis, 38th FEBS Congress. FEBS Journal. http://dx.doi.org/10.1111/febs.12340 | 8 |
| Yi Q, Liu O, Yan F, Lin X, Diao S, Wang L, Jin L, Wang S, Lu Y, Fan Z. Analysis of Senescence-Related Differentiation Potentials and Gene Expression Profiles in Human Dental Pulp Stem Cells. Cells Tissues Organs. 2017;203(1):1-11. doi: 10.1159/000448026. Epub 2016 Sep 15. PMID: 27627434. | 5 |
| Ambrosi TH, Goodnough LH, Steininger HM, Hoover MY, Kim E, Koepke LS, Marecic O, Zhao L, Seita J, Bishop JA, Gardner MJ, Chan CKF. Geriatric fragility fractures are associated with a human skeletal stem cell defect. Aging Cell. 2020 Jul;19(7):e13164. doi: 10.1111/acel.13164. Epub 2020 Jun 14. PMID: 32537886; PMCID: PMC7370785. | 5 |
| Das M, Das A, Barui A, Paul RR. Comparative evaluation of proliferative potential and replicative senescence associated changes in mesenchymal stem cells derived from dental pulp and umbilical cord. Cell Tissue Bank. 2022 Mar;23(1):157-170. doi: 10.1007/s10561-021-09926-8. Epub 2021 Apr 26. PMID: 33900487. | 4 |
| Cheng Y, Lin KH, Young TH, Cheng NC. The influence of fibroblast growth factor 2 on the senescence of human adipose-derived mesenchymal stem cells during long-term culture. Stem Cells Transl Med. 2020 Apr;9(4):518-530. doi: 10.1002/sctm.19-0234. Epub 2019 Dec 16. PMID: 31840944; PMCID: PMC7103622. | 4 |

**Exclusion Criteria**

1. Studies that evaluate the impact of senescence on other cell types (e.g., somatic cells, fibroblasts, or epithelial cells).
2. Studies that evaluate the impact of senescence in disease models unrelated to regenerative therapies (e.g., cancer, aging-related diseases, etc.).
3. Studies using immortalized/cancer cell lines or genetically modified cells (e.g., knock-in/knock-out for senescence-related genes).
4. Studies without a direct comparison between non-senescent and senescent groups.
5. Studies that do not confirm the senescent state of the cells.
6. Studies that focus solely on the mechanistic pathways of senescence without assessing its direct impact on tissue repair or regenerative therapies.
7. Full paper copy not available or inaccessible.
8. Reviews articles, systematic reviews, meta-analyses, abstracts, protocols, short communications, personal opinions, letters, posters, conference abstracts, and case report.

**SUPPLEMENTARY FIGURE 1 Risk of bias assessment of** in vitro **studies.** Studies were appraised using the PETRICCS-based tool. Bars represent the percentage of items rated as “Yes” (low risk), “No” (high risk), or “Unclear” (some concerns).

**SUPPLEMENTARY FIGURE 2 Risk of bias assessment of** in vivo **studies.** Studies were appraised using the SYRCLE’S tool. Bars represent the percentage of items rated as “Yes” (low risk), “No” (high risk), or “Unclear” (some concerns).

**SUPPLEMENTARY TABLE 3** Individual results for the proliferation outcome.

| Author | Senescence Induction Method | Stem Cell Type | Nº of replicates (n) | Assay | NS (mean ± SD or SEM) | S (mean ± SD or SEM) | Significance (p-value) | Lower Proliferative Potential | Notes |  |  |
| --- | --- | --- | --- | --- | --- | --- | --- | --- | --- | --- | --- |
| Feng *et al*., 2014 [11] | Inflammatory Stimuli | DPSCs | 3 | BrdU assay (370 nm)  Cell Number (10^3^) | ~0.89 ± 0.1  ~65.13 ± 5.9 | ~0.37 ± 0.04  ~34.97 ± 3.06 | p<0.05  p<0.05 | Senescent | S: LPS 10 ng/mL, 6x |  |  |
| Feng *et al*., 2018 [18] | Inflammatory Stimuli | DPSCs | See (Feng *et al*., 2014)a | | | | | **-** | Proliferation data identical of Feng *et al.,* 2014. |  |  |
| Alt *et al*., 2012 [19] | Chronological Aging | AD-MSCs | 8 | PDT (hours) | ~46.19 ± 8.14 | ~62.67 ± 3.87 | p<0.01 | Senescent | The CFU assay showed a significantly higher colony-forming percentage in the young group. |  |  |
| Choudhery *et al*., 2014 [9] | Chronological Aging | AD-MSCs | 3 | PDT (hours) | 62.0 ± 5.9 | 89.1 ± 26.6 | p<0.05 | Senescent | CFU assay showed ~30% reduced colony formation in aged donors; quantitative values could not be extracted. TPD was also assessed, with higher values observed in the younger group. |  |  |
| Gruber *et al*., 2012 [10] | Chronological Aging | AD-MSCs | 3 | Mean DT (hours) | ~55.56 ± 4.2 | ~89.41 ± 5.84 | p<0.0001 | Senescent | S: 40-75 years |  |  |
| Kornicka *et al*., 2015 [20] | Chronological Aging | AD-MSCs | 3 | PDT (hours) | 55 ± 9.8 | ~130 ± 5.5 | p<0.01 | Senescent | S: >70 years. CFU-E assay was performed, but quantitative data were not extracted for synthesis; however, the aged group showed a lower percentage of CFU-E. |  |  |
| Legzdina *et al*., 2016 [44] | Chronological Aging | AD-MSCs | 2-3 | CPD | 28.97* | 23.28* | not significant | not significant | NS: CS-8 (27 years), S: CS-3 (63 years). Cells from eight donors showed pronounced donor-dependent heterogeneity in proliferation kinetics (PD/PDT). CFU and MTT assays and PDT curves were performed, with no significant differences observed between younger and older donors. |  |  |
| Wu *et al*., 2022 [21] | Chronological Aging | AD-MSCs | 3 | PDT (days) | ~1 ± 0.03 | ~1.26 ± 0.09 | p<0.01 | Senescent | S: adult. A growth curve assay was performed; however, quantitative data were not extracted for synthesis. The fold increases in the cell numbers of the infant were significantly higher than those of the adult. |  |  |
| Gao *et al*., 2023 [58] | Chronological Aging | BM-MSCs | 3 | Ki-67 (%) | ~49.78 ±4.08  ~11.42* | ~9.92 ± 1.9  ~6.33* | p<0.001 | Senescent | The CPD analysis revealed that the proliferative capacity was exhausted by passage 7 in the aged group, while remaining active until at least passage 12 in the young group. |  |  |
| Huang *et al*., 2023 [22] | Chronological Aging | BM-MSCs | 3 | Ki-67 (%) | ~74.15 ± 2.25 | ~23.66 ± 2.09 | NR | Senescent | NS: Lenti-Control (fig.:2C), S: Control-siRNA (fig.: 3C). Groups were not directly compared. |  |  |
| Katsube *et al*., 2008 [27] | Chronological Aging | BM-MSCs | 4 | Growth Rate (fold increase) | ~20* | ~5* | NR | NR | NS: “High” (1 year), S: “Low” (74 years). |  |  |
| Stolzing *et al*., 2008 [28] | Chronological Aging | BM-MSCs | NR | CPD | ~32.25* | ~21.69* | NR | NR | NS: mean of two 18-year-old donors; S: mean of two 50-year-old donors. Cell cultures from donors ≤9 years old exhibited early proliferative arrest (CPD). CFU-f assay was performed and a significant decline was seen in the older age group (p=0.007). |  |  |
| Wagner *et al*., 2008 [29] | Chronological Aging | BM-MSCs | NR | CPD | ~9.06* | ~7.84* | NR | NR | NS: donor 7 (25 years), S: mean of 44- and 42-years old donors. |  |  |
| Chen *et al*., 2024 [23] | Chronological Aging | DPSCs | 3 | CCK-8 (450 nm) | ~2.28* | ~1.79* | p<0.001 | Senescent | Data were extracted from day 9. |  |  |
| Feng *et al*., 2014 [24] | Chronological Aging | DPSCs | 3 | BrdU (370 nm) | ~0.87 ± 0.15 | ~0.32± 0.05 | p<0.05 | Senescent | NS: 5-12 years, S: >50 years. Growth curve was performed and after day 4, cells from younger group exhibited more rapid proliferation rates compared to other groups. These results indicated that the capacity of proliferation was gradually inhibited by age. |  |  |
| Horibe *et al*., 2014 [12] | Chronological Aging | DPSCs | 6 | Cumulative Cell Number | ~10^12^* | ~10^11^* | NR | NR | - |  |  |
| Iezzi *et al*., 2019 [25] | Chronological Aging | DPSCs | 3 | PDT (days) | ~4.84 ± 0.48 | ~13.95 ± 1.06 | p<0.05 | Senescent | S: Group C (62-66 years). |  |  |
| Wang *et al*., 2025 [26] | Chronological Aging | DPSCs | 5 | CCK-8 (450 nm) | ~3.23 ± 0.18 | ~1.29 ± 0.13 | p<0.001 | Senescent | Data were extracted from day 7. |  |  |
| Christodoulou *et al*., 2013 [33] | Serial Passaging | AD-MSCs | NR | PDT (hours) | ~210 ± 55,38 | ~623.07 ± 83.08 | NR | NR | NS: P3, S: P10. CPD and Proliferation Index was performed and exhibited severely limited proliferative capacity, with an early plateau in cumulative PD and a rapid decline in proliferation index after passage 2. |  |  |
| Danisovic *et al*., 2017 [30] | Serial Passaging | AD-MSCs | 5 | Proliferation Index (%) | ~40.72 ± 0.43 | ~34.78 ± 0.26 | p<0.01 | Senescent | NS: P1, S: P30. The proliferation index increased until P10, slightly decreased, and remained stable until P25; by P30, proliferative activity was reduced to 32%. |  |  |
| Figiel-Dabrowska *et al*., 2022 [57] | Serial Passaging | AD-MSCs | 4-6 | PDT (days) | ~3.81 ± 0.37 | ~13.53 ± 0.03 | NR | NR | NS: P3, S: P20. The study evaluated different O₂ concentrations, however only data from the 21% O₂ group (standard culture conditions) were extracted for analysis. Data from the 5% O₂ group were documented but excluded as an experimental intervention outside the scope of this review. Additional assays were performed but focused on different O₂ concentrations. |  |  |
| Gruber *et al*., 2012 [10] | Serial Passaging | AD-MSCs | 3 | Mean DT (hours) | ~55.56 ± 4.2 | ~89.07 ± 10.63 | NR | NR | NS: P1-P4, S: P5-P11. |  |  |
| Ma *et al*., 2019 [14] | Serial Passaging | AD-MSCs | 5 | Cell Count (10^5^) | ~10.16* | ~18.14* | NR | NR | NS: P3, S: P6. Data from day 3. |  |  |
| Wu *et al*., 2022 [21] | Serial Passaging | AD-MSCs | 3 | PDT (days) | ~1 ± 0.03  ~1.26 ± 0.09 | ~1.48 ± 0.04  ~2.67 ± 0.51 | NR | NR | NS: P3; S: P10. Infant and adult donors. Growth curve analysis showed higher fold increases in cell number at early versus late passages. |  |  |
| Zhang *et al*., 2024 [13] | Serial Passaging | AD-MSCs | 4 | BrdU (360 nm) | ~0.25 ± 0.008 | ~0.21 ± 0.006 | p<0.05 | Senescent | NS: P4, S: P10. |  |  |
| Bertolo *et al.*, 2016 [42] | Serial Passaging | BM-MSCs | 7 | PDT (hours) | ~4,309.63 ± 376.57 | ~7,405.85 ± 1,317.99 | p<0.05 | Senescent | NS: P3; S: P10. P3 vs P9 (p<0.05). CPD and CFU assays were performed, revealing marked inter-donor variability in cumulative population doublings and CFU counts, with progressive decline in colony numbers from P3 to P11. |  |  |
| Bonab *et al.*, 2006 [47] | Serial Passaging | BM-MSCs | NR | PD (passage) | 2.6 ± 0.68 | P10: 1.2 ± 0.73 | not significant | not significant | NS: P3; S: P10. CPD analysis was performed and revealed a gradual decline in proliferative potential. After 120 days in culture, proliferative potential decreased more rapidly. |  |  |
| Kim *et al*., 2021 [35] | Serial Passaging | BM-MSCs | NR | PDT (days) | LD: 1.3±0.02  MD: 1.9±0.03  HD: 1.8±0.05 | LD: 3.4±0.10  MD: 5.6±0.82  HD: 22.2±3.48 | NR | NR | NS: P5, S: P15. The acronyms LD, MD, and HD refer to Low-Density (50 cells/cm²), Medium-Density (1,000 cells/cm²), and High-Density (4,000 cells/cm²) cultures, respectively. |  |  |
| Ma *et al*., 2019 [14] | Serial Passaging | BM-MSCs | 5 | Cell Count (10^5^) | ~6.94* | ~7.96* | NR | NR | NS: P3, S: P6. Data from day 3. |  |  |
| Wenjing *et al*., 2024 [6] | Serial Passaging | BM-MSCs | 3 | MTT (490 nm) | ~0.743 | ~0.447 | NR | NR | NS: P5, S: P15. Data from day 7. |  |  |
| Diomede *et al*., 2017 [43] | Serial Passaging | DPSCs | 3 | MTT (650 nm) | ~18,666.45 ± 444.45 | ~17.925.92 ± 641.98 | not significant | not significant | NS: P2; S: P15. Data from day 7. The Trypan Blue exclusion test was performed and revealed no statistically significant differences between passages. |  |  |
| Ma *et al*., 2019 [14] | Serial Passaging | DPSCs | 5 | Cell Count (10^5^) | ~27.79 ± 2.04 | ~37.38* | NR | NR | NS: P3, S: P6. Data from day 3. |  |  |
| Diomede *et al*., 2017 [43] | Serial Passaging | PDLSCs | 3 | MTT (650 nm) | ~10,447.59 ± 425.77 | ~9661.57 ± 425.76 | not significant | not significant | NS: P2; S: P15. Data from day 7. The Trypan Blue exclusion test was performed and revealed no statistically significant differences between passages. |  |  |
| Hu *et al*., 2024 [31] | Serial Passaging | PDLSCs | 3 | CCK-8 (450 nm) | ~0.43 ± 0.03 | ~0.27 ± 0.02 | p<0.001 | Senescent | NS: P4, S: P20. Data from day 7. |  |  |
| Jia *et al*., 2025 [32] | Serial Passaging | PDLSCs | 3 | CCK-8 (450 nm) | ~3.2* | ~0.31 ± 0.05 | p<0.001 | Senescent | NS: P5, S: P20. Data from day 6. |  |  |
| Ma *et al*., 2019 [14] | Serial Passaging | PDLSCs | 5 | Cell Count (10^5^) | ~20.67 ± 2.71 | ~27.87 ± 1.77 | NR | NR | NS: P3, S: P6. Data from day 3. |  |  |
| Yang *et al*., 2021 [40] | Serial Passaging | PDLSCs | 3 | CCK-8 (450 nm) | ~2.46 ± 0.1 | ~1.5* | NR | NR | NS: 3, S: P12. Data from day 7. |  |  |
| Christodoulou *et al*., 2013 [33] | Serial Passaging | UC-MSCs-WJ | NR | PDT (hours) | ~34.46 ± 7.5 | ~38.98 ± 6.25 | NR | NR | NS: P3, S: P10. CPD and Proliferation Index analysis demonstrated a high and sustained proliferative capacity, with continuous accumulation of PD over at least 15 passages and a peak Proliferation Index at passage 6. |  |  |
| Zhuang *et al*., 2015 [36] | Serial Passaging | UC-MSCs-WJ | 3 | MTT (570 nm) | ~0.64 ± 0.04 | ~0.42 ± 0.04 | NR | NR | NS: P3, S: P15. Data from day 6. |  |  |
| Jin *et al*., 2016 [34] | Serial Passaging | UC-MSCs-CB | 2 | Fold Increase | MSC1: ~1 ± 0.03  MSC2: ~1 ± 0.1  MSC3: ~1 ± 0.0 | MSC1: ~0.34 ± 0.04  MSC2: ~0.22 ± 0.03  MSC3: ~0.37 ± 0.09 | NR | NR | NS: P5, S: P13. |  |  |
| Kim *et al*., 2020 [60] | Serial Passaging | UC-MSCs-CB | 4 | PD | ~4.05 ± 1.3 | ~1.74 ± 0.46 | NR | NR | NS: P3, S: P14. Cells were grouped by size (heterogeneous, large, and small); the reported data refer to the heterogeneous group. |  |  |

**Supplementary Table 3. Individual results for the proliferation outcome.** This table summarizes individual study results evaluating proliferation-related outcomes in non-senescent (NS) and senescent (S) stem cell populations. Studies are first grouped according to the type of senescence induction (inflammatory stimuli, Chronological aging, and serial passaging) and then sub-grouped by cell type (AD-MSCs, BM-MSCs, DPSCs, PDLSCs, UC-MSCs-WJ and UC-MSCs-CB). Data are presented as mean ± SD or SEM, as reported in the original studies. Different assays were used to assess proliferative capacity, including BrdU incorporation, cell number, population doublings (PD), doubling time (DT), population doubling time (PDT), cumulative population doublings (CPD), and colony-forming unit (CFU) assays. When more than one assay was used to assess the same outcome, only one was included in the table. NR indicates not reported. Values preceded by a tilde (~) indicate data extracted manually from figures when numerical results were not reported in the text. An asterisk (*) indicates that SD or SEM was not reported in the original study. In some studies, NS and S groups represent different experimental controls (e.g., Lenti-Control or Control-siRNA) and were not directly compared.

**SUPPLEMENTARY TABLE 4** Individual results for the osteogenic differentiation outcome.

| Author | Senescence Induction Method | Stem Cell Type | Nº of replicates (n) | Assay | NS (mean ± SD or SEM) | S (mean ± SD or SEM) | Significance (p-value) | Lower Differentiation Potential | Notes |
| --- | --- | --- | --- | --- | --- | --- | --- | --- | --- |
| Ma *et al*., 2019 [14] | Inflammatory Stimuli – LPS and TNF- α | BM-MSCs | 5 | Alizarin Red (area - %) | ~69.38 ± 5.28 | LPS: ~25.33 ± 2.32  TNF-α: ~22.24 ± 1.76 | NR | NR | LPS: 1 µg/mL  TNF-α: 100 ng/mL  2 weeks. |
| Ma *et al*., 2019 [14] | Inflammatory Stimuli – LPS and TNF- α | DPSCs | 5 | Alizarin Red (area - %) | ~84.36 ± 8.15 | LPS: ~39.64 ± 4.41  TNF-α: ~19.82 ± 3.52 | NR | NR | LPS: 1 µg/mL  TNF-α: 100 ng/mL  2 weeks. |
| Alt *et al*., 2012 [19] | Chronological Aging | AD-MSCs | 5 | ALP Concentration (ng) | ~48.73 ± 1.66 | ~21.34 ± 1.18 | NR | NR | NS: group 1 (<20 years), S: group 3 (>50 years). 12 days. Significantly decreased with age; although the p-value was not reported, this finding was described in the results. |
| Choudhery *et al*., 2014 [9] | Chronological Aging | AD-MSCs | 3 | Von Kossa staining (% positive area) | 20.0% ± 1.7 | 8.9% ± 2.2 | p<0.001 | Senescent | NS: group 1 (<30 years), S: group 3 (>60 years). Similar results were obtained when gene expression of OST and ALP was analyzed through quantitative RT-PCR. Induction time was not reported. |
| Kornicka *et al*., 2015 [20] | Chronological Aging | AD-MSCs | ≥2 | SEM/EDX (nº of bone nodules) | ~42.82 ±2.46 | ~4.51 ± 1.18 | p<0.001 | Senescent | NS: >20 years, S: >70 years. 16 days. Cells from young donors exhibited greater osteogenic outcomes, including increased calcium and phosphorus deposition in the extracellular matrix, enhanced formation of mineralized nodules, higher ALP activity, and elevated expression of osteogenic markers (such as *OPN* and *OCN*). In contrast, these osteogenic features were progressively reduced in cells isolated from older donors. |
| Legzdina *et al*., 2016 [44] | Chronological Aging | AD-MSCs | NR | Alizarin Red S staining  (Calcium deposits) | positive | positive | NR | NR | NS: CS-8 (27 years), S: CS-8 (63 years). 30 days. Alkaline phosphatase activity was positive in both age groups. |
| Wu *et al*., 2013 [37] | Chronological Aging | AD-MSCs | NR | Calcium content (µmol/µg) | ~6.41 ± 0.59 | ~4.0.3 ± 0.13 | p<0.05 | Senescent | NS: infant, S: adult. Data are shown for day 21; days 7 and 14 were also evaluated. Osteogenic differentiation decreased with age, with higher mineralized matrix deposition and *RUNX-2* expression in infant donors and reduced calcium phosphate content in elderly donors. Paracrine osteoinductive factor expression, including *BMP-2* and *TGF-β*, was preserved across ages. |
| Wu *et al*., 2022 [21] | Chronological Aging | AD-MSCs | 3 | RT-qPCR - *OCN* | ~1.96 ± 0.51 | ~27.14 ± 3.08 | p<0.001 | Non-Senescent | NS: Infant, S: Adult. 7 days. *RUNX* expression was higher in the adult group. |
| Ye *et al*., 2016 [38] | Chronological Aging | AD-MSCs | 6 | Alizarin Red Staining (% positive area) | 86.7 ± 10.0 | 65.1 ± 15.1 | p<0.05 | Senescent | 2 weeks. Alizarin Red S solubilization (OD 562 nm) revealed a significant reduction in the S group (p < 0.01). |
| Stolzing *et al*., 2008 [28] | Chronological Aging | BM-MSCs | NR | ALP activity (nmol p-NP/min/10⁴ cells) | 1.45 ± 0.1 | 0.54 ± 0.04 | p<0.001 | Senescent | 10 days. |
| Chen *et al*., 2024 [23] | Chronological Aging | DPSCs | NR | Alizarin Red S staining | positive | reduced | NR | NR | 21 days. No quantitative analysis was performed. |
| Feng *et al*., 2014 [24] | Chronological Aging | DPSCs | 3 | Alizarin Red Staining (OD) | ~1 ± 0.08 | ~0.29 ± 0.04 | p<0.05 | Senescent | 21 days. |
| Horibe *et al*., 2014 [12] | Chronological Aging | DPSCs | 6 | Alizarin Red S staining | positive | positive | Not significant | not-significant | 28 days. *OCN* expression assessed by RT-PCR was similar in both groups. |
| Iezzi *et al*., 2019 [25] | Chronological Aging | DPSCs | 3 | Alizarin Red Staining (540 nm) | ~5.40 ± 0.29 | ~2.32 ± 0.13 | p<0.05 | Senescent | NS: group A (21 years), S: group C (64 years). 14 days. 3 weeks. Reduced ALP staining and significantly decreased osteogenesis-related gene expression were observed in aged group (*RUNX2, BGLAP, BMP2, β-catenin, DMP1, DSPP*). |
| Wang *et al*., 2025 [26] | Chronological Aging | DPSCs | NR | Alizarin red S staining | positive | reduced | NR | NR | 7 days. |
| Christodoulou *et al*., 2013 [33] | Serial Passaging | AD-MSCs | 3 | Alizarin Red Staining | >50 nodules | <20 nodules | NR | NR | NS: P2, S: P10. 21 days. Early passage showed robust osteogenic capacity assessed by calcium deposition, and CFU-ALP assays, whereas late passage (P10) cells exhibited reduced osteogenic outcomes. |
| Gruber *et al*., 2012 [10] | Serial Passaging | AD-MSCs | NR | Alizarin Red Staining | positive | positive | Not significant | not-significant | NS: P1, S: P12. No quantitative analysis was performed; however, both passages analyzed tested positive for osteogenic potential by forming mineralized nodules in vitro. Induction time was not reported. |
| Legzdina *et al*., 2016 [44] | Serial Passaging | AD-MSCs | NR | Alizarin Red S staining  (Calcium deposits) | positive | reduced | NR | NR | NS: P3, S: P9. CS-8 donor (27 years) Alkaline phosphatase activity was positive in both passages. Induction time was not reported. |
| Ma *et al*., 2019 [14] | Serial Passaging | AD-MSCs | 5 | Alizarin Red (area - %) | ~65.26 ± 9.92 | ~43.92 ± 1.73 | p≤0.01 | Senescent | NS: P3, S: P6. 2 weeks. |
| Truong, *et al*., 2019 [45] | Serial Passaging | AD-MSCs | NR | Alizarin Red staining | positive | reduced | NR | NR | NS: P5, S: P15. 30 days. |
| Wang *et al*., 2025 [46] | Serial Passaging | AD-MSCs | 3 | Alizarin Red Staining (405 nm) | ~2.77 ± 0.89 | ~3.12 ± 0.18 | NR | NR | NS: P4, S: P8. 3 weeks. |
| Bertolo *et al.*, 2016 [42] | Serial Passaging | BM-MSCs | 7 | RT-PCR - *OPN* | ~1 ± 0.01 | ~0.58 ± 0.63 | Not significant | not-significant | NS: P3. S: P11. 21 days. Mineralized matrix deposition assessed by von Kossa staining was higher in early passages and decreased with in vitro aging, with significant inter-donor variability (P3 vs P9). |
| Bonab *et al.*, 2006 [47] | Serial Passaging | BM-MSCs | NR | Von Kossa and ALP (%) | 100 | 20 | NR | NR | NS: P1-4, S: P10. 2-3 weeks. |
| Cheng *et al*., 2011 [39] | Serial Passaging | BM-MSCs | 3 | Alizarin Red (595 nm) | ~0.32 ± 0.03 | ~0.54 ± 0.04 | p<0.01 | Senescent | Ns: P4, S: P12. 21 days. *ALPL* expression was higher in late passage group. |
| Kim *et al*., 2021 [35] | Serial Passaging | BM-MSCs | NR | Alizarin Red (405 nm) | LD: ~0.49 ± 0.05  MD: ~0.54 ± 0.1  HD: ~0.48 ± 0.03 | LD: ~0.23 ± 0.03  MD: ~0.24 ± 0.02  HD: ~0.11 ± 0.02 | NR | NR | NS: P5, S: P15. Alizarin Red S mineralization decreased in a passage-dependent manner, with reduced *ALPP* and *SPP1* in late passage; no direct statistical test between P5 and P15 was performed (described in text). LD, MD, and HD denote low-, medium-, and high-density cultures. Induction time was not reported. |
| Ma *et al*., 2019 [14] | Serial Passaging | BM-MSCs | 5 | Alizarin Red (area - %) | ~83.63 ± 6.94 | ~52.60 ± 6.95 | p≤0.01 | Senescent | NS: P3, S: P6. 2 weeks. |
| Wagner *et al*., 2008 [29] | Serial Passaging | BM-MSCs | NR | Alizarin Red Staining (595 nm) | ~0.22 ± 0.02 | ~0.42 ± 0.13 | NR | NR | NS: P3, S: P11. 21 days. Von Kossa Staining showed higher mineralization in late passage. |
| Wenjing *et al*., 2024 [6] | Serial Passaging | BM-MSCs | 3 | ALP Staining (Positive Cell Rate-%) | 94.1 ± 3.3 | 82.4 ± 8.7 | Not significant | not-significant | NS: P5, S: P15. Induction time was not reported. |
| Diomede *et al*., 2017 [43] | Serial Passaging | DPSCs | 2 | qRT-PCR - *ALP* | ~0.9 ± 0.05 | ~1.1 ± 0.08 | Not significant | not-significant | NS: P2, S: P15. 28 days. *RUNX* expression was assessed and showed higher levels in early passage; however, no significant difference was observed. Alizarin Red S staining revealed mineralized nodules in both passages. |
| Li *et al*., 2025 [61] | Serial Passaging | DPSCs | 3 | Alizarin Red Staining (562 nm) | ~2.24 ± 0.34 | ~0.66 ± 0.12 | p<0.001 | Senescent | NS: P3, S: P12. 21 days. |
| Ma *et al*., 2019 [14] | Serial Passaging | DPSCs | 5 | Alizarin Red (area - %) | ~80.14 ± 5.21 | ~71.46 ± 5.71 | Not significant | not-significant | NS: P3, S: P6. 2 weeks. |
| Diomede *et al*., 2017 [43] | Serial Passaging | PDLSCs | 2 | qRT-PCR - *ALP* | ~0.98 ± 0.04 | ~1.06 ± 0.09 | Not significant | not-significant | *RUNX* expression was assessed and showed higher levels in late passage; however, no significant difference was observed. Alizarin Red S staining revealed mineralized nodules in both passages. |
| Jia *et al*., 2025 [32] | Serial Passaging | PDLSCs | 3 | ALP Activity Assay (U/mg protein) | ~81.94* | ~14.85 ± 0.96 | p<0.001 | Senescent | NS: P5, S: P20. 21 days. *COL1, ALP, and RUNX2* expression was assessed and showed decreased levels at late passages, along with fewer mineralized nodules observed in the Alizarin Red S staining assay. |
| Ma *et al*., 2019 [14] | Serial Passaging | PDLSCs | 5 | Alizarin Red (area - %) | ~70.22 ± 6.2 | ~55.83 ± 3.97 | Not significant | not-significant | NS: P3, S: P6. |
| Yang *et al*., 2021 [40] | Serial Passaging | PDLSCs | 3 | Alizarin Red S Staining (562 nm) | ~1 ± 0.09 | ~0.46 ± 0.07 | p<0.01 | Senescent | NS: P3, S: P12. 28 days. ALP activity was reduced, as indicated by quantitative assessment and weaker staining. Expression of osteogenic genes (*ALP, RUNX2, OCN, COL-I*) was lower compared with late passage. |
| Cheng *et al*., 2011 [39] | Serial Passaging | UC-MSCs-WJ | NR | Alizarin Red (595 nm) | ~0.43 ± 0.03 | ~1.18 ± 0.03 | p<0.01 | Non-Senescent | NS: P4, S: P23. 21 days. *ALPL* expression was higher in late passage group. |
| Christodoulou *et al*., 2013 [33] | Serial Passaging | UC-MSCs-WJ | 3 | Alizarin Red Staining | >50 nodules | <20 nodules | NR | NR | NS: P2, S: P20. 21 days. Early passage showed robust osteogenic capacity assessed by calcium deposition, and CFU-ALP assays, whereas late passage (P20) cells exhibited reduced osteogenic outcomes. |
| Govarthanan *et al*., 2025 [41] | Serial Passaging | UC-MSCs-WJ | 3 | RT-qPCR - *OPN* | ~8.09 ± 0.57 | ~2.03 ± 0.11 | p<0.0001 | Senescent | NS: P3, S: P15. 21 days. *COL1A1, RUNX2* expression were assessed and showed reduced levels in late passage, along with fewer mineralized nodules observed in the Alizarin Red S staining assay. |
| Scheers *et al*., 2013 [48] | Serial Passaging | UC-MSCs-WJ | 5 | Alizarin Red Staining (bone matrix deposition area - %) | ~91.36 ± 1.36 | ~1.36 ± 0.45 | NR | NR | NS: P3, S: P21. A significant difference was observed between P9 and P21 (p < 0.001). The percentage of area with bone matrix deposition was similar at passages 3, 9, and 18. |
| Jin *et al*., 2016 [34] | Serial Passaging | UC-MSCs-CB | NR | ALP staining | positive | reduced | NR | NR | NS: P5, S: P13. 14 days. |

**Supplementary Table 4. Individual results for the osteogenic differentiation outcome.** This table summarizes individual study results evaluating osteogenic differentiation capacity in non‑senescent (NS) and senescent (S) mesenchymal stem cell populations. Studies are grouped by senescence induction method (inflammatory stimuli, Chronological aging, serial passaging) and sub‑grouped by cell type (AD‑MSCs, BM‑MSCs, DPSCs, PDLSCs, UC‑MSCs-WJ, UC‑MSCs-CB). Data are presented as mean ± SD or SEM, as reported in the original studies. A variety of osteogenic assays were used, including Alizarin Red staining (area, optical density), von Kossa staining, alkaline phosphatase (ALP) activity, calcium content, RT‑qPCR of osteogenic markers (e.g., *OCN, OPN, RUNX2*), and others. When multiple assays were reported for the same outcome, only one representative assay was included. NR indicates “not reported”. Values preceded by a tilde (~) indicate data extracted manually from figures when numerical results were not reported in the text. An asterisk (*) denotes that SD or SEM was not provided in the original article.

**SUPPLEMENTARY TABLE 5** Individual results for the adipogenesis differentiation outcome.

| Author | Senescence Induction Method | Stem Cell Type | Nº of replicates (n) | Assay | NS (mean ± SD or SEM) | S (mean ± SD or SEM) | Significance (p-value) | Lower Differentiation Potential | Notes |
| --- | --- | --- | --- | --- | --- | --- | --- | --- | --- |
| Alt *et al*., 2012 [19] | Chronological Aging | AD-MSCs | 5 | Oil Red O Staining  (% positive cells) | ~33.29 ± 2.6 | ~9.78 ± 2.21 | NR | NR | NS: group 1 (<20 years), S: group 3 (>50 years). 12 days. Reduced *LPL* and *CEBPA* expression and increased *LEP* expression were observed in the elderly group. |
| Choudhery *et al*., 2014 [9] | Chronological Aging | AD-MSCs | 3 | Oil Red O staining (% positive cells) | 67.4% ± 7.1% | 62.2% ± 13.0% | Not significant | not significant | NS: group 1 (<30 years), S: group 3 (>60 years). 21 days. *PPAR-ү, LPL* expression showed no difference between groups. |
| Kornicka *et al*., 2015 [20] | Chronological Aging | AD-MSCs | ≥2 | Oil Red O positive area (%) | ~21.53 ± 1.95 | ~39.27 ± 2.93 | p<0.01 | Non-Senescent | NS: >20 years, S: >70 years. 14 days. *LPL* and *CEBPA* expression were reduced and *LEP* expression was increased in the senescent group. Lipid droplet formation was higher at early time points and similar between groups by day 14. |
| Legzdina *et al*., 2016 [44] | Chronological Aging | AD-MSCs | NR | Oil Red O staining | positive | positive | NR | NR | NS: CS-8 (27 years), S: CS-8 (63 years). 16 days. |
| Wu *et al*., 2022 [21] | Chronological Aging | AD-MSCs | NR | RT-qPCR - *LPL* | ~42.25 ± 3.59 | ~75.56 ± 9.35 | p<0.01 | Non-Senescent | NS: Infant, S: Adult. 14 days. *PPARγ* expression was increased in adult group. |
| Ye *et al*., 2016 [38] | Chronological Aging | AD-MSCs | 6 | Oil Red O Staining (% positive cells) | ~78.7% ± 9.8 | ~59.4% ± 12.7 | p<0.05 | Senescent | 14 days. *PPARγ2* and *LPL* expression were significantly reduced in old group. |
| Stolzing *et al*., 2008 [28] | Chronological Aging | BM-MSCs | NR | Oil Red O Staining (% positive cells) | 66% ± 12 | 59% ± 9.5 | Not significant | not significant | NS: young (7-18 years), S: aged (>40 years). |
| Feng *et al*., 2014 [24] | Chronological Aging | DPSCs | 3 | Oil Red O staining (OD) | ~0.76 ± 0.08 | ~0.16 ± 0.04 | p<0.05 | Senescent | 3 weeks. |
| Horibe *et al*., 2014 [12] | Chronological Aging | DPSCs | 6 | Oil red O staining | positive | positive | Not significant | not significant | *PPARγ* and *aP2* expression were similar in both groups. |
| Iezzi *et al*., 2019 [25] | Chronological Aging | DPSCs | NR | Oil Red O staining | positive | reduced | NR | NR | NS: group A (21 years), S: group C (64 years). 14 days. |
| Legzdina *et al*., 2016 [44] | Serial Passaging | AD-MSCs | NR | Oil Red O staining | positive | reduced | NR | NR | NS: P3, S: P9. CS-8 donor (27 years). 16 days. |
| Truong, *et al*., 2019 [45] | Serial Passaging | AD-MSCs | NR | Oil Red O staining | positive | positive | NR | NR | NS: P5, S: P15. 7 days. Qualitative analyses showed almost no difference. |
| Wang *et al*., 2025 [46] | Serial Passaging | AD-MSCs | 3 | Oil red staining (450 nm) | positive | reduced | NR | NR | NS: P4, S: P8. 2 weeks. Qualitative analyses showed dramatically decayed in late passage. |
| Bertolo *et al.*, 2016 [42] | Serial Passaging | BM-MSCs | 7 | RT-qPCR *- PPAR-ɣ* | ~0.98 ± 0.08 | ~6.44 ± 1.66 | p<0.05 | Non-Senescent | NS: P3, S: P11. 3 weeks*.* Qualitative analysis of Oil Red O staining showed increased lipid accumulation at late passages. |
| Bonab *et al.*, 2006 [47] | Serial Passaging | BM-MSCs | NR | Staining with Oil red O (lipid vacuoles – %) | 100 | 40 | NR | NR | NS: PQ-P4, S: P9. 2-3 weeks. |
| Cheng *et al*., 2011 [39] | Serial Passaging | BM-MSCs | 3 | Oil Red-O staining (490 nm) | ~0.65 ± 0.06 | ~0.2 ± 0.05 | p<0.01 | Senescent | NS: P4, S: P12. 21 days. *CEBPα, PPARRɣ* expression was significantly higher at early passages. |
| Kim *et al*., 2021 [35] | Serial Passaging | BM-MSCs | NR | Oil Red O (500 nm) | LD: ~0.98 ± 0.05  MD: ~1.05 ± 0.14  HD: ~1.05 ± 0.14 | LD: ~0.55 ± 0.03  MD: ~0.48 ± 0.05  HD: ~0.53 ± 0.05 | NR | NR | NS: P5, S: P15. Oil Red O staining decreased in a passage-dependent manner; however, no direct statistical test between P5 and P15 was performed (described in the text). *PPARγ* and *FABP4* expression appeared similar across passages. LD, MD, and HD denote low-, medium-, and high-density cultures. Induction time was not reported. |
| Wagner *et al*., 2008 [29] | Serial Passaging | BM-MSCs | NR | Oil Red (595 nm) | ~0.48 ± 0.02 | ~0.17 ± 0.14 | NR | NR | NS: P3, S: P11. 21 days. |
| Wenjing *et al*., 2024 [6] | Serial Passaging | BM-MSCs | 3 | Oil Red O Staining (Positive Cell Rate - %) | 40.8 ± 2.0 | 34.2 ± 1.8 | Not significant | not significant | NS: P5, S: P15. Induction time was not reported. |
| Diomede *et al*., 2017 [43] | Serial Passaging | DPSCs | 2 | RT-qPCR - *PPAR-ɣ* | ~1 ± 0.06 | ~1.06 ± 0.08 | Not significant | not significant | NS: P2, S: P15. 28 days. Oil red solution staining demonstrates the presence of lipid vacuoles at cytoplasmic level in both passages. *FABP4* expression showed no difference. |
| Diomede *et al*., 2017 [43] | Serial Passaging | PDLSCs | 2 | RT-qPCR - *PPAR-ɣ* | ~1.31 ± 0.1 | ~1.32 ± 0.12 | Not significant | not significant | NS: P2, S: P15. 28 days. Oil red solution staining demonstrates the presence of lipid vacuoles at cytoplasmic level in both passages. *FABP4* expression showed no difference. |
| Cheng *et al*., 2011 [39] | Serial Passaging | UC-MSCs-WJ | 3 | Oil Red-O staining (490 nm) | ~0.27 ± 0.06 | ~0.11 ± 0.04 | p<0.01 | Senescent | NS: P4, S: P12. 21 days. *CEBPα* increased at late passages, while *PPARγ* was higher at early passages. |
| Scheers *et al*., 2013 [48] | Serial Passaging | UC-MSCs-WJ | 5 | Oil Red O staining (% of cells containing lipid droplets) | ~69.23 ± 2.41 | ~7.9 ± 2.64 | NR | NR | NS: P3, S: P21. A significant difference was observed between P9 and P21 (p < 0.001). The percentage of area with lipid droplets was similar at passages 3, 9, and 18. |
| Jin *et al*., 2016 [34] | Serial Passaging | UC-MSCs-CB | NR | Oil Red O staining | positive | reduced | NR | NR | NS: P5, S: P13. Induction time was not reported. |

**Supplementary Table 5. Individual results for the adipogenic differentiation outcome.** This table summarizes individual study results evaluating adipogenic differentiation capacity in non‑senescent (NS) and senescent (S) mesenchymal stem cell populations. Studies are grouped by senescence induction method (Chronological aging, serial passaging) and sub‑grouped by cell type (AD‑MSCs, BM‑MSCs, DPSCs, PDLSCs, UC‑MSCs-WJ, UC‑MSCs-CB). Data are presented as mean ± SD or SEM, as reported in the original studies. Adipogenic assays included Oil Red O staining (positive area, optical density, percentage of positive cells) and RT‑qPCR of adipogenic markers (e.g., *PPARγ, LPL, CEBPA*). When multiple assays were reported for the same outcome, only one representative assay was included. NR indicates “not reported”. Values preceded by a tilde (~) indicate data extracted manually from figures when numerical results were not reported in the text. An asterisk (*) denotes that SD or SEM was not provided in the original article.

**SUPPLEMENTARY TABLE 6** Individual results for the chondrogenic differentiation outcome.

| Author | Senescence Induction Method | Stem Cell Type | Nº of replicates (n) | Assay | NS (mean ± SD or SEM) | S (mean ± SD or SEM) | Significance (p-value) | Lower Differentiation Potential | Notes |
| --- | --- | --- | --- | --- | --- | --- | --- | --- | --- |
| Alt *et al*., 2012 [19] | Chronological Aging | AD-MSCs | 5 | RT-PCR – *COL2A* | baseline | -3±0.4 | NR | NR | S: group 3 (>50 years). 21 days. *BMP-6* and *COL10A* expression were reduced in aged group. |
| Choudhery *et al*., 2014 [9] | Chronological Aging | AD-MSCs | 3 | Alcian Blue staining (620 nm) | 0.51 ± 0.06 | 0.17 ± 0.02 | p<0.001 | Senescent | S: group 3 (>60 years). 3 weeks. *AGGRECAN* and *COL2* expression were significantly reduced in aged group. |
| Wu *et al*., 2022 [21] | Chronological Aging | AD-MSCs | NR | Alcian Blue – diameter of pellets (mm) | ~2.01 ± 0.06 | ~2.47 ± 0.13 | p<0.05 | Non-Senescent | NS: infant, S: adult. Day 21. *SOX9, COL2* and *COL10* expression were significantly reduced in adult group. |
| Ye *et al*., 2016 [38] | Chronological Aging | AD-MSCs | 6 | Alcian Blue staining (620 nm) | 0.76 ± 0.29 | 0.25 ± 0.16 | p<0.05 | Senescent | 3 weeks. AGGRECAN and *COL2* were significantly reduced in aged group. |
| Stolzing *et al*., 2008 [28] | Chronological Aging | BM-MSCs | 5 | Alcian blue binding assay (630 nm) | 3.2 ± 3 | 1.6 ± 1.9 | Not significant | not significant | S: aged. 21 days. |
| Iezzi *et al*., 2019 [25] | Chronological Aging | DPSCs | NR | Alcian Blue staining | positive | positive | Not significant | not significant | S: group C (64 years). 14 days. |
| Gruber *et al*., 2012 [10] | Serial Passaging | AD-MSCs | NR | Alcian Blue staining | positive | positive | Not significant | not significant | NS: P1, S: P12. No quantitative analysis was performed; however, both passages analyzed tested positive for chondrogenic potential by forming micromass formation *in vitro*. Induction time was not reported. |
| Truong, *et al*., 2019 [45] | Serial Passaging | AD-MSCs | NR | Safranin O staining | positive | reduced | NR | NR | NS: P5, S: P15. 21 days. |
| Wang *et al*., 2025 [46] | Serial Passaging | AD-MSCs | NR | Alcian Blue staining | positive | reduced | NR | NR | NS: P4, S: P8. 14 days |
| Bertolo *et al.*, 2016 [42] | Serial Passaging | BM-MSCs | 3 | GAG/DNA (µg/µg) | ~66.01 ± 9.91 | ~5.82 ± 4.47 | p<0.05 | Senescent | NS: P3, S: P11. 28 days. *COL2* were significantly reduced in late passage. |
| Wenjing *et al*., 2024 [6] | Serial Passaging | BM-MSCs | 3 | Safranin O Staining (Positive Cell Rate) | 94.3 ± 6.5 | 84.5 ± 8.5 | Not significant | not significant | NS: P5, S: P15. Induction time was not reported. |
| Govarthanan *et al*., 2025 [41] | Serial Passaging | UC-MSCs-WJ | 3 | RT-qPCR – *COL2A1* | ~10.13 ± 0.39 | ~2.62 ± 0.22 | p<0.0001 | Senescent | NS: P3, S: P15. 28 days. *SOX9* expression was significantly reduced at late passage, whereas *COL1A1* expression was similar in both passages. Toluidine Blue staining was reduced in the late passage. |

**Supplementary Table 6. Individual results for the chondrogenic differentiation outcome.** This table summarizes individual study results evaluating chondrogenic differentiation capacity in non‑senescent (NS) and senescent (S) mesenchymal stem cell populations. Studies are grouped by senescence induction method (Chronological aging, serial passaging) and sub‑grouped by cell type (AD‑MSCs, BM‑MSCs, DPSCs, UC‑MSCs-WJ). Data are presented as mean ± SD or SEM, as reported in the original studies. Chondrogenic assays included Alcian Blue staining, Safranin O staining, glycosaminoglycan/DNA (GAG/DNA) quantification, pellet diameter measurement, and RT‑qPCR of chondrogenic markers (e.g., *COL2A1, SOX9, AGGRECAN*). When multiple assays were reported for the same outcome, only one representative assay was included. Values preceded by a tilde (~) indicate data manually extracted from figures. NR indicates “not reported”.

**SUPPLEMENTARY TABLE 7** Individual results for the migration outcome.

| Author | Senescence Induction Method | Stem Cell Type | Nº of replicates (n) | Assay | NS (mean ± SD or SEM) | S (mean ± SD or SEM) | Significance (p-value) | Reduced Migration Potential | Notes |
| --- | --- | --- | --- | --- | --- | --- | --- | --- | --- |
| Chen *et al*., 2024 [23] | Chronological Aging | DPSCs | 3 | Transwell | ~173.04 ± 18.64 | ~42.54 ± 4.3 | p<0.001 | Senescent | S: old group. 24 hours. |
| Wenjing *et al*., 2024 [6] | Serial Passaging | BM-MSCs | 3 | Transwell | 182.3 ± 18.3 | 19.2 ± 5.0 | p<0.05 | Senescent | NS: P5, S: P15. 24 hours. Scratch Healing Rate was assessed and showed reduced migration in late passage. |
| Yang *et al*., 2021 [40] | Serial Passaging | PDLSCs | 3 | Transwell (relative number of migrated cells) | ~1 ± 0.06 | ~0.39 ± 0.03 | p<0.01 | Senescent | NS: P3, S: P12. 48 hours. |

**Supplementary Table 7. Individual results for the migration outcome.** This table summarizes individual study results evaluating migratory capacity in non‑senescent (NS) and senescent (S) mesenchymal stem cell populations. Studies are grouped by senescence induction method (Chronological aging, serial passaging) and sub‑grouped by cell type (DPSCs, BM‑MSCs, PDLSCs). Data are presented as mean ± SD or SEM, as reported in the original studies. Migration was assessed using Transwell assays (number of migrated cells) and scratch‑wound healing assays. Values preceded by a tilde (~) indicate data manually extracted from figures. NR indicates “not reported”.

**SUPPLEMENTARY TABLE 8** Individual results for the immune response outcome.

| Author | Senescence Induction Method | Stem Cell Type | Nº of replicates (n) | Assay | NS (mean ± SD or SEM) | S (mean ± SD or SEM) | Significance (p-value) | Notes |
| --- | --- | --- | --- | --- | --- | --- | --- | --- |
| Huang *et al*., 2023 [22] | Chronological Aging | BM-MSCs | 3 | Elisa (pg/mL) | TNF-α: ~29.26 ± 4.18  IL-6: ~30.31 ± 2.61  IL-10: ~107.14 ± 4.7 | TNF-α: ~105.1 ± 1.59  IL-6: ~116.19 ± 7.92  IL-10: ~63.90 ± 4.76 | NR | NS: Lenti-Control (YMSCs), S: Control-siRNA (AMSCs). Groups were not directly compared. |
| Horibe *et al*., 2014 [12] | Serial Passaging | DPSCs | 6 | RT-PCR | *IL-1β*: ~1.5  *IL-6*: ~2.2  *IL-8*: ~2.5  *Groα*: ~2.1 | *IL-1β*: ~3.2  *IL-6*: ~5.2  *IL-8*: ~6.1  *Groα*: ~4.8 | NR | NS: P6, S: P20. |
| Zhuang *et al*., 2015 [36] | Serial Passaging | UC-MSCs^1^ | 3 | RT-qPCR | baseline: 1 | *IDO-1*: ~1.02 ± 0.12  *HMOX-1*: ~1.59 ± 0.14  *IL-10*: ~1.56 ± 0.11  *IL-6*: ~1.52 ± 0.12  *iNOS*: ~1.42 ± 0.13  *IL-1ɑ*: ~0.59 ± 0.1  *TGF-β1*: ~0.98 ± 0.08  *IL-1β*: ~0.53 ± 0.14  *IFN-ɣ*: ~0.44 ± 0.08 | p<0.05  except: *IDO-1* and *TGF-β1* | NS: P3, S: P15. |
| Yu *et al*., 2014 [49] | Serial Passaging | UC-MSCs^2^ | 3 | Elisa – PGE_2_ (pg/mL) | 1. 400.48 ± 109.22 2. 436.89 ± 109.22 | 1. 364.07 ± 109.23 2. 327.66 ± 109.23 | NR | NS: P5-10, S: P15-P20. 1 and 2 represent independent donor-derived hMSC lines (#1 and #2). |

**Supplementary Table 8. Individual results for the immune response outcome.** This table summarizes individual study results evaluating immune‑modulatory marker expression in non‑senescent (NS) and senescent (S) mesenchymal stem cell populations. Studies are grouped by senescence induction method (Chronological aging, serial passaging) and sub‑grouped by cell type (BM‑MSCs, DPSCs, UC‑MSCs-WJ, UC‑MSCs-CB). Data are presented as mean ± SD or SEM, as reported in the original studies. Immune‑related markers were assessed by ELISA (cytokine secretion) or RT‑qPCR (gene expression) for factors including *TNF‑α, IL‑6, IL‑10, IL‑1β, IL‑8, Groat, IDO‑1, HMOX‑1, iNOS, TGF‑β1, IFN‑γ*, and *PGE₂*. Values preceded by a tilde (~) indicate data manually extracted from figures. NR indicates “not reported.”

**SUPPLEMENTARY TABLE 9** Individual results for the tissue repair (*in vivo)* outcome.

| Author | Senescence Induction Method | Stem Cell Type | Nº of replicates (n) | Assay | NS (mean ± SD or SEM) | S (mean ± SD or SEM) | Significance (p-value) | Lower Tissue Repair | Notes |
| --- | --- | --- | --- | --- | --- | --- | --- | --- | --- |
| Ma *et al*., 2019 [14] | Inflammatory Stimuli – LPS and TNF- α | BM-MSCs | 5 | HE staining (mineralization area - %) | ~0.93 ± 0.16 | ~0.53 ± 0.49 | NR | NR | Mineralization in periodontal tissue. Nude mice. LPS: 1µg/mL  TNF-α: 100ng/mL |
| Ma *et al*., 2019 [14] | Inflammatory Stimuli – LPS and TNF- α | DPSCs | 5 | HE staining (mineralization area - %) | ~0.68 ± 0.11 | ~0.59 ± 0.08 | NR | NR | Mineralization in periodontal tissue. Nude mice. LPS: 1µg/mL  TNF-α: 100ng/mL |
| Chen *et al*., 2024 [23] | Chronological Aging | DPSCs | 6 | Micro-CT analysis (bone mineral density g/cc) | ~0.56 ± 0.05 | ~0.44 ± 0.04 | NR | NR | Bone regeneration (rat calvarial bone defects). Sprague-Dawley rats. |
| Horibe *et al*., 2014 [12] | Chronological Aging | DPSCs | 4 | Vascularization area/regenerated area | ~9.17 ± 0.98 | ~7.21 ± 0.41 | p<0.05 | Senescent | Aged DPSCs exhibited reduced pulp regeneration, with smaller regenerated pulp tissue and fewer odontoblast-like cells compared with young DPSCs. |
| Yang *et al*., 2021 [40] | Serial Passaging | PDLSCs | 3 | HE staining (mineralization) | positive | reduced | NR | NR | NS: P3, S: P12. Nude mice. |

**Supplementary Table 9. Individual results for the tissue repair (*in vivo*) outcome.** This table summarizes individual study results evaluating *in vivo* tissue repair capacity of non‑senescent (NS) and senescent (S) mesenchymal stem cell populations. Studies are grouped by senescence induction method (inflammatory stimuli, Chronological aging, serial passaging) and sub‑grouped by cell type (BM‑MSCs, DPSCs, PDLSCs). Data are presented as mean ± SD or SEM, as reported in the original studies. *In vivo* repair outcomes were assessed using micro‑CT analysis (bone mineral density) and histomorphometry (HE staining; mineralization area). Values preceded by a tilde (~) indicate data manually extracted from figures. NR indicates “not reported”.
